# Electrochemical vicinal oxyazidation of α-arylvinyl acetates

**DOI:** 10.3762/bjoc.18.103

**Published:** 2022-08-12

**Authors:** Yi-Lun Li, Zhaojiang Shi, Tao Shen, Ke-Yin Ye

**Affiliations:** 1 Institute of Pharmaceutical Science and Technology, College of Chemistry, Fuzhou University, Fuzhou 350108, China; 2 Frontiers Science Center for Transformative Molecules, School of Chemistry and Chemical Engineering, Shanghai Jiao Tong University, Shanghai 200240, China

**Keywords:** azide, azidoketone, electrosynthesis, enol acetate, radical

## Abstract

α-Azidoketones are valuable and versatile building blocks in the synthesis of various bioactive small molecules. Herein, we describe an environmentally friendly and efficient electrochemical vicinal oxyazidation protocol of α-arylvinyl acetates to afford diverse α-azidoketones in good yields without the use of a stoichiometric amount of chemical oxidant. A range of functionality is shown to be compatible with this transformation, and further applications are demonstrated.

## Introduction

Organoazides play important roles in pharmaceutical, bioorthogonal chemistry, and many other interdisciplinary research areas [1–3]. Among them, azidoketones are also very versatile building blocks in organic synthesis, pharmaceutical, and materials science [4–6]. Therefore, the development of a green, efficient, and sustainable protocol for the synthesis of azidoketones is of great significance [7–9].

Retrosynthetically, the nucleophilic substitution of α-bromoketones by sodium azide [10] and oxidation of the azido alcohols [11] are among the most straightforward methods to generate azidoketones. Remarkably, photoredox [12] and electrochemical [13] oxyazidation of vinylarenes are also becoming competent synthetic approaches. Besides vinylarenes, vinyl acetates are potentially versatile precursors for the anticipated vicinal oxyazidation.

**Scheme 1:**
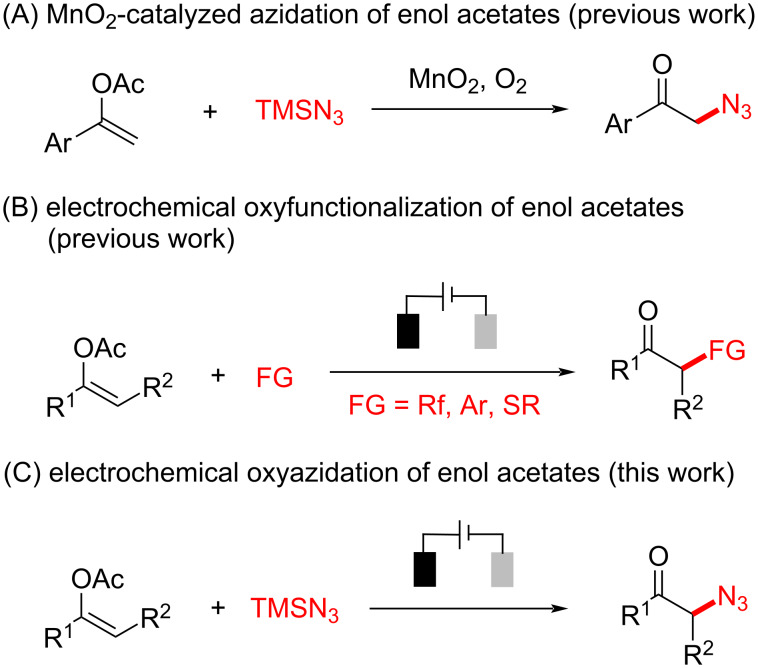
From vinyl acetates to α-azidoketones.

For instance, Singh and co-workers have reported a manganese dioxide-catalyzed radical azidation of enol acetates to afford the corresponding azidoketones using dioxygen as the oxidant (Scheme 1A) [14]. The adoption of electrosynthesis in green and sustainable redox transformations has been experiencing a dynamic renaissance [15–22] not only because it employs the passage of charge instead of chemical oxidants or reductants but also offers opportunities for the precise control of reactivity by "dialing-in" the specific potential on demand [23–25].

Specifically, the electrochemical oxyfunctionalization of vinyl acetates has been developed to afford the corresponding α-fluorinated [26], -arylated [27], and -sulfenylated [28] ketones (Scheme 1B). In addition, electrochemical azidation [29–33] has also become a robust and reliable synthetic tool to incorporate azido functionality [34–35] into diverse organic frameworks. Herein, we report that the electrochemical oxyfunctionalization strategy could be well applied to the synthesis of α-azidoketone using readily available α-arylvinyl acetates, and azidotrimethylsilane (Scheme 1C).

## Results and Discussion

The constant cell potential electrolysis (*E*_cell_ = 2.3 V, carbon cloth anode, and Pt cathode) of 1-phenylvinyl acetate (**1**) with azidotrimethylsilane was performed and the desired α-azidoketone (**2**) was obtained in 68% yield (Table 1, entry 1, for details of the reaction optimization see Supporting Information File 1). The cyclic voltammetry studies showed while there was no obvious oxidation peak for TMSN_3_, 1-phenylvinyl acetate (**1**) exhibits two oxidation peaks. The first peak (*E*_p/2_ = 1.51 V vs Fc^+/0^) was assigned to be the oxidation of the vinyl acetate moiety. The control experiment demonstrated that there was no conversion without an electric current (Table 1, entry 2). In addition, in the absence of the electrolyte only a low yield of the desired product was obtained (Table 1, entry 3). The use of *n*-Bu_4_NPF_6_ was crucial because lower yields were generally obtained with other electrolytes, such as *n*-Bu_4_NOAc or *n*-Bu_4_NBF_4_ (Table 1, entries 4 and 5). Note that the yield decreased without the addition of water suggesting water may facilitate the formation of the azidoketone (Table 1, entry 6). Interestingly, the H_2_^18^O labeling experiment confirmed that there was no ^18^O incorporation in the obtained α-azidoketone (**2**, for details see Supporting Information File 1). Therefore, the oxygen source of the newly formed carbonyl moiety may originate directly from the vinyl acetate. This conclusion is also consistent with the fact that even in the absence of water, the desired α-azidoketone was still obtained.

**Table 1:** Optimization of reaction conditions.^a^

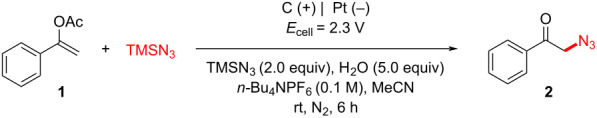		

Entry	Variation from the standard reaction conditions	Yield (%)

1	none	65 (68)^b^
2	no current	n.d.
3	without electrolyte	9
4	*n*-Bu_4_NOAc instead of *n*-Bu_4_NPF_6_	12
5	*n*-Bu_4_NBF_4_ instead of *n*-Bu_4_NPF_6_	35
6	without H_2_O	34

^a^Reaction conditions: **1** (0.5 mmol), TMSN_3_ (1.0 mmol), *n*-Bu_4_NPF_6_ (0.5 mmol), H_2_O (2.5 mmol), MeCN (5 mL), carbon cloth anode, platinum cathode, undivided cell, *E*_cell_ = 2.3 V, room temperature, 6 h, yields were determined using ^1^H NMR with dibromomethane as the internal standard. ^b^Isolated yield.

**Scheme 2:**
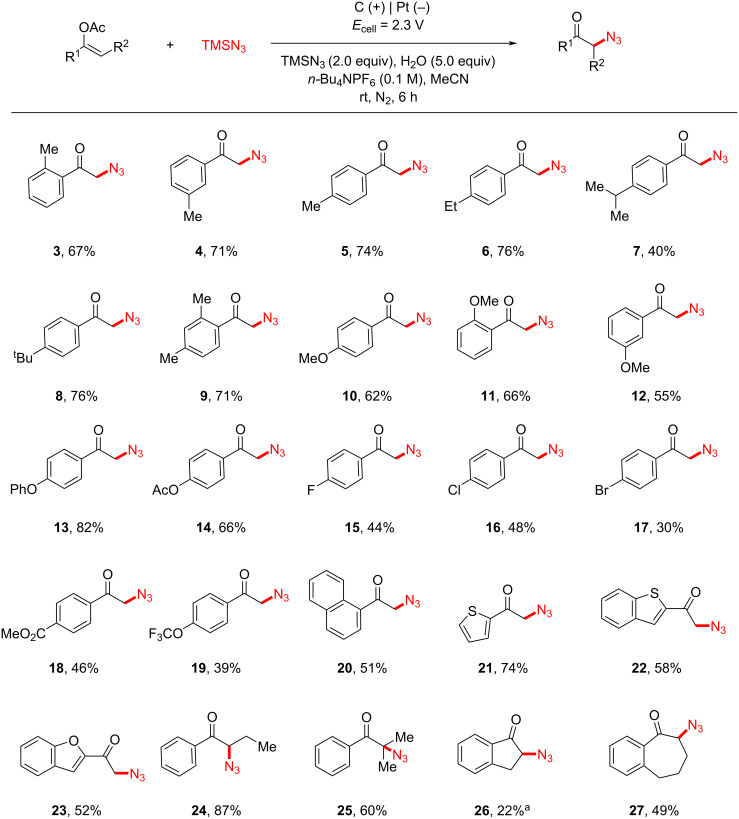
Substrate scope. Reaction conditions: α-arylvinyl acetate (0.5 mmol), TMSN_3_ (1.0 mmol), *n*-Bu_4_NPF_6_ (0.5 mmol), H_2_O (2.5 mmol), MeCN (5 mL), carbon cloth anode, platinum cathode, undivided cell, *E*_cell_ = 2.3 V, room temperature, 6 h. ^a^2 h.

Under the optimal conditions, the substrate scope of this electrochemical oxyazidation reaction was investigated (Scheme 2). Enol acetates derived from various alkyl-substituted phenylacetones were generally well tolerated (**3**–**9**, 40–76% yields). The relatively low yield of the isopropyl-substituted one (**7**) was attributed to the competing oxidation of the isopropylbenzene moiety [31,36], which was consistent with its relative low oxidation potential (*E*_onset_ = 1.68 V vs Fc^+/0^). Other electron-donating substituents, such as the OMe (**10**–**12**), OPh (**13**), and OAc (**14**) were also well tolerated.

**Scheme 3:**
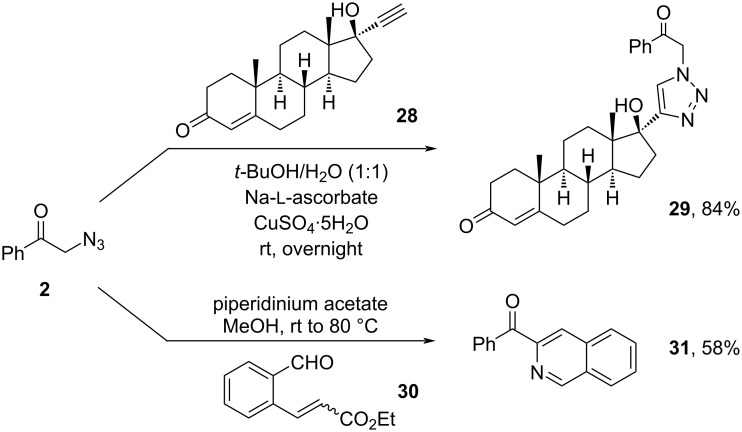
Derivatization of α-azidoketone **2**.

However, halogenated substrates, including fluoro (**15**), chloro (**16**), and bromo (**17**), proceeded with the anticipated reactivity less efficiently (30–48% yields). The presence of other electron-withdrawing groups, such as CO_2_Me (**18**) and OCF_3_ (**19**), exhibited similar negative effects on the reaction yields. Naphthalene (**20**), thiophene (**21**), benzothiophene (**22**), and benzofuran (**23**) were all amenable in this transformation. In addition, various linear- (**24**, **25**) and cyclic enol acetates (**26**, **27**) also readily underwent the anticipated oxyazidation. Unfortunately, the current protocol was not applicable to the oxyazidation of enol acetate deriving from aliphatic ketones, such as cyclohexanone (see Supporting Information File 1 for details). As illustrated in Scheme 3, the synthetic utility of α-azidoketone was further evaluated [37–38]. Click reaction between 2-azido-1-phenylethan-1-one (**2**) and ethisterone (**28**) [39–41] readily afforded the target triazole product (**29**) in 84% yield. Upon treatment with piperidinium acetate and ethyl 3-(2-formylphenyl)acrylate (**30**), α-azidoketone (**2**) was transformed into isoquinoline product (**31**) in 58% yield [42].

**Scheme 4:**
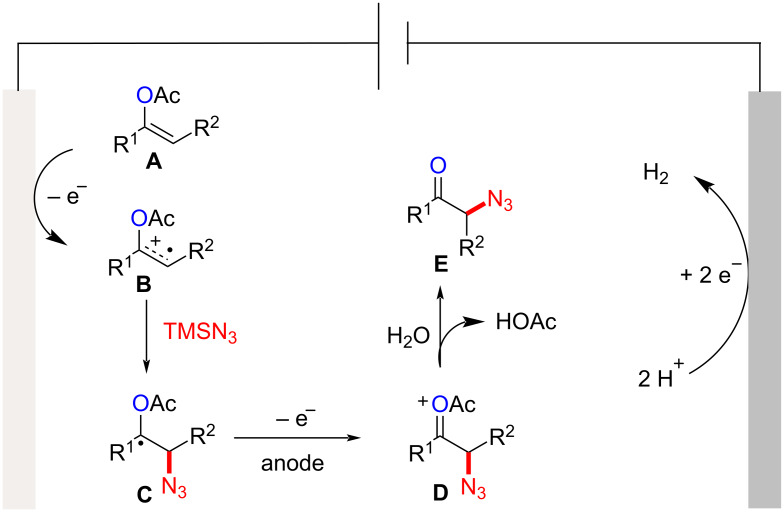
Proposed mechanism.

Based on our reaction results and the known literature [13–14], a possible mechanism is proposed (Scheme 4). The enol acetate **A** first undergoes anodic oxidation to form a radical cation intermediate **B**, which is then intercepted by azidotrimethylsilane to afford the benzyl radical **C**. Subsequently, this radical is further anodically oxidized to its oxocarbenium ion intermediate **D**, which finally reacts with water to form the desired product **E**. According to our ^18^O labeling experiment, the oxygen source of the newly installed carbonyl group probably originates from the vinyl acetate, not from H_2_O.

## Conclusion

In summary, we have developed an environmentally friendly and efficient electrochemical oxyazidation of α-arylvinyl acetates to access diverse vicinal α-azidoketones. The protocol employs the experimentally simple undivided electrochemical cell and tolerates a broad substrate scope. The obtained α-azidoketones have been shown to be versatile building blocks for the preparation of biologically relevant heterocycles.

## Supporting Information

File 1 Experimental procedures, characterization data, copies of ^1^H and ^13^C NMR spectra.

## References

[1] Sletten E M, Bertozzi C R (2011). Acc Chem Res.

[2] Li J, Chen P R (2016). Nat Chem Biol.

[3] Prescher J A, Bertozzi C R (2005). Nat Chem Biol.

[4] Kádár Z, Frank É, Schneider G, Molnár J, Zupkó I, Kóti J, Schönecker B, Wölfling J (2012). ARKIVOC.

[5] Brusis T, Grofe H J, Förster D, Weuta H (1977). Infection (Munich, Ger).

[6] Zhao R, Chen B-C, Bednarz M S, Wang B, Skoumbourdis A P, Sundeen J E, Dhar T G M, Iwanowicz E J, Balasubramanian B, Barrish J C (2007). ARKIVOC.

[7] Erythropel H C, Zimmerman J B, de Winter T M, Petitjean L, Melnikov F, Lam C H, Lounsbury A W, Mellor K E, Janković N Z, Tu Q (2018). Green Chem.

[8] Frontana-Uribe B A, Little R D, Ibanez J G, Palma A, Vasquez-Medrano R (2010). Green Chem.

[9] Kim Y, Li C-J (2020). Green Synth Catal.

[10] Patonay T, Hoffman R V (1994). J Org Chem.

[11] Yang B, Lu Z (2017). ACS Catal.

[12] Hossain A, Vidyasagar A, Eichinger C, Lankes C, Phan J, Rehbein J, Reiser O (2018). Angew Chem, Int Ed.

[13] Ye Z, Zhu R, Wang F, Jiang H, Zhang F (2021). Org Lett.

[14] Panday P, Garg P, Singh A (2018). Asian J Org Chem.

[15] Yan M, Kawamata Y, Baran P S (2017). Chem Rev.

[16] Kingston C, Palkowitz M D, Takahira Y, Vantourout J C, Peters B K, Kawamata Y, Baran P S (2020). Acc Chem Res.

[17] Francke R, Little R D (2014). Chem Soc Rev.

[18] Yoshida J-i, Shimizu A, Hayashi R (2018). Chem Rev.

[19] Xiong P, Xu H-C (2019). Acc Chem Res.

[20] Jiao K-J, Xing Y-K, Yang Q-L, Qiu H, Mei T-S (2020). Acc Chem Res.

[21] Novaes L F T, Liu J, Shen Y, Lu L, Meinhardt J M, Lin S (2021). Chem Soc Rev.

[22] Shi S-H, Liang Y, Jiao N (2021). Chem Rev.

[23] Aoyama M, Fukuhara T, Hara S (2008). J Org Chem.

[24] Lam K, Markó I E (2009). Org Lett.

[25] Yu Y, Jiang Y, Wu S, Shi Z, Wu J, Yuan Y, Ye K (2022). Chin Chem Lett.

[26] Vil’ V A, Merkulova V M, Ilovaisky A I, Paveliev S A, Nikishin G I, Terent’ev A O (2021). Org Lett.

[27] de Souza A A N, Bartolomeu A d A, Brocksom T J, Noël T, de Oliveira K T (2022). J Org Chem.

[28] Zhou P, Liu Y, Xu Y, Wang D (2022). Org Chem Front.

[29] Wu J, Dou Y, Guillot R, Kouklovsky C, Vincent G (2019). J Am Chem Soc.

[30] Meyer T H, Samanta R C, Del Vecchio A, Ackermann L (2021). Chem Sci.

[31] Niu L, Jiang C, Liang Y, Liu D, Bu F, Shi R, Chen H, Chowdhury A D, Lei A (2020). J Am Chem Soc.

[32] Siu J C, Parry J B, Lin S (2019). J Am Chem Soc.

[33] Fu N, Sauer G S, Saha A, Loo A, Lin S (2017). Science.

[34] Wu K, Liang Y, Jiao N (2016). Molecules.

[35] Ge L, Chiou M-F, Li Y, Bao H (2020). Green Synth Catal.

[36] Sharma A, Hartwig J F (2015). Nature.

[37] Bangalore P K, Vagolu S K, Bollikanda R K, Veeragoni D K, Choudante P C, Misra S, Sriram D, Sridhar B, Kantevari S (2020). J Nat Prod.

[38] Lv G, Qiu L, Li K, Liu Q, Li X, Peng Y, Wang S, Lin J (2019). New J Chem.

[39] Buil M L, Esteruelas M A, Garcés K, Oñate E (2009). Organometallics.

[40] Levine P M, Lee E, Greenfield A, Bonneau R, Logan S K, Garabedian M J, Kirshenbaum K (2012). ACS Chem Biol.

[41] Levine P M, Imberg K, Garabedian M J, Kirshenbaum K (2012). J Am Chem Soc.

[42] Prasad B, Phanindrudu M, Tiwari D K, Kamal A (2019). J Org Chem.

