# Supplementary material for: Electrochemical vicinal oxyazidation of α-arylvinyl acetates
Source: Beilstein J Org Chem. 2022 Aug 12;18:1026–31. doi: 10.3762/bjoc.18.103 (PMC9379640; doi:10.3762/bjoc.18.103)
Supplement: File 1 — Experimental procedures, characterization data, copies of 1H and 13C NMR spectra. [file Beilstein_J_Org_Chem-18-1026-s001.pdf]

BEILSTEIN JOURNAL OF ORGANIC CHEMISTRY

Supporting Information

for

Electrochemical vicinal oxyazidation of α-arylvinyl acetates

Yi-Lun Li, Zhaojiang Shi, Tao Shen and Ke-Yin Ye

Beilstein J. Org. Chem. **2022,** *18,* 1026–1031. doi:10.3762/bjoc.18.103

Experimental procedures, characterization data, copies of ^{1}H and ^{13}C NMR spectra

License and Terms: This is a supporting information file under the terms of the Creative Commons Attribution License (https://creativecommons.org/licenses/by/4.0). Please note that the reuse, redistribution and reproduction in particular requires that the author(s) and source are credited and that individual graphics may be subject to special legal provisions.
The license is subject to the *Beilstein Journal of Organic Chemistry* terms and conditions: (https://www.beilstein-journals.org/bjoc/terms)

Table of Contents

1. GENERAL INFORMATION S2

2. OPTIMIZATION OF REACTION CONDITIONS S4

3. GENERAL PROCEDURES S6

4. CHARACTERIZATION OF PRODUCTS S7

5. UNSUCCESSFUL SUBSTRATES S15

6. DERIVATIZATIONS OF THE PRODUCT S15

7. MECHANISM STUDIES S16

7.1 CYCLIC VOLTAMMETRY STUDIES **S16**

7.2 ^{18}O LABELLING EXPERIMENT **S18**

8. REFERENCES S20

9. SPECTRAL DATA (^{1}H, ^{13}C, ^{19}F) OF PRODUCTS S21

1. General Information

All reactions were performed under an atmosphere of nitrogen using standard undivided three-necked glassware unless otherwise indicated. All commercial reagents were used without further purification unless otherwise noted. Reactions were monitored by thin-layer chromatography (TLC) analysis. TLC plates were viewed under UV light. Yields refer to products isolated after purification by column chromatography unless otherwise stated. Proton nuclear magnetic resonance (^{1}H NMR) spectra, carbon nuclear magnetic resonance (^{13}C NMR) spectra, and fluorine nuclear magnetic resonance (^{19}F NMR) were recorded on Bruker AV-400 (400 MHz) and JEOL-500 (500 MHz) spectrometers. Chemical shifts for protons are reported in parts per million downfield from tetramethylsilane and are referenced to residual protium in the NMR solvent ($CHCl_3$ = δ 7.26). Chemical shifts for carbon are reported in parts per million downfield from tetramethylsilane and are referenced to the carbon resonances. IR spectra were obtained from Thermo Scientific NICOLET 380 FT-IR. HRMS were obtained on an Exactive Plus LC-MS (ESI) mass spectrometer with the use of a quadrupole analyzer. Cyclic voltammetry data were measured with a CHI 760E potentiostat (Chinstruments). All chemicals were purchased from *TCI Shanghai* or *Energy Chemical* and used as received.

CAUTION: Organic azides are known to be potentially explosive compounds. While we did not encounter any issues during their synthesis, proper precautions were taken. All azidation reactions and subsequent workups should be performed behind a blast shield. Once isolated, organic azides should be stored below room temperature and away from sources of heat, light, pressure and shock.

Electrolysis experiments were performed using the MESTEK DC power supply. Electrode clips (PT-1 or PT-3) and platinum plate (99.99%, 15 × 15 × 0.3 mm, or 30 × 30 × 0.1 mm) were purchased from Gaoss Union. The carbon cloth (CeTech WOS1002) was cut into 15 × 15 × 0.1 mm pieces before use and was clamped between electrode clips.

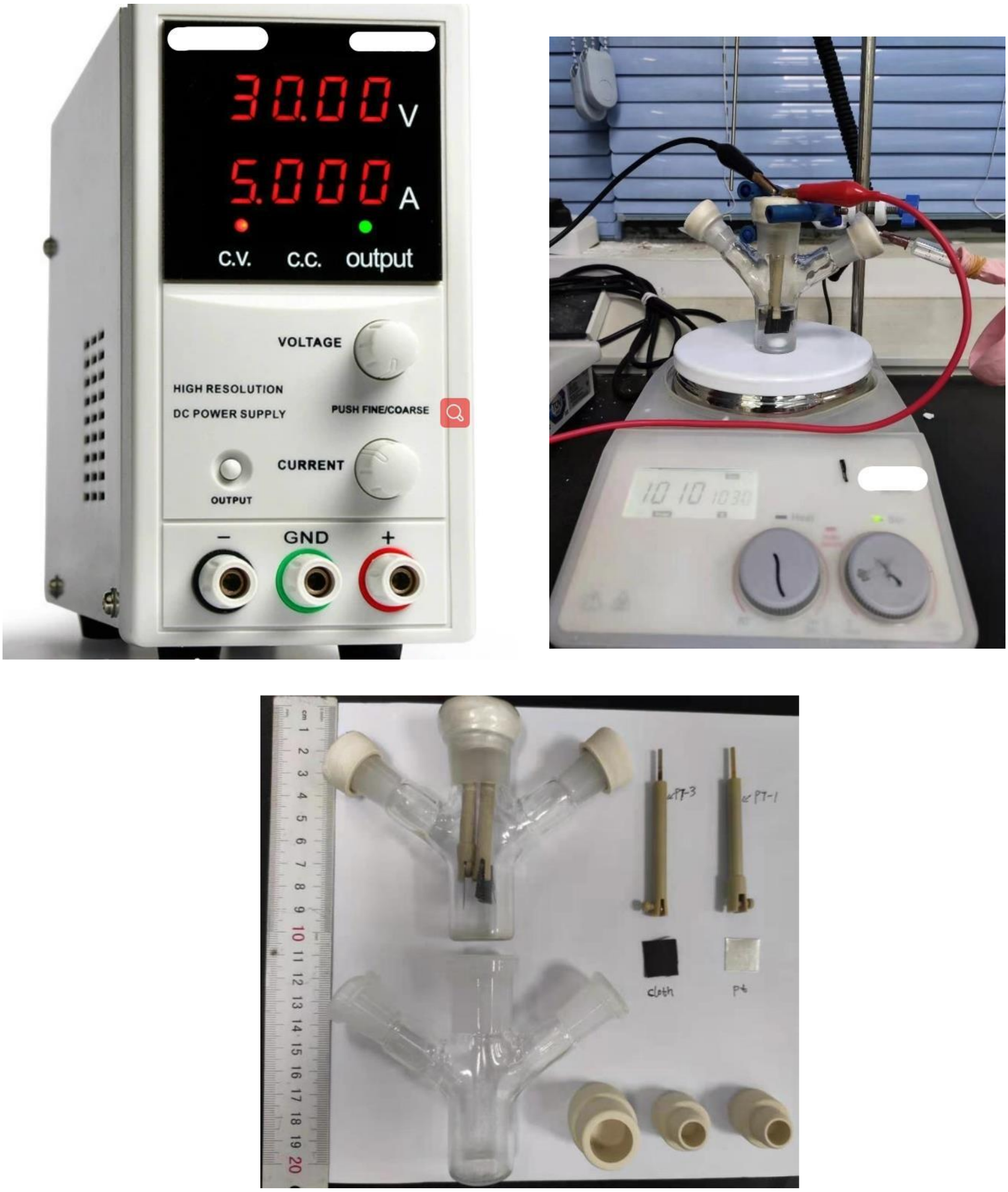

Figure S1: Electrolysis setups (We hereby confirm that all three photos depicted in **Figure S1** were taken by my co-authors and have not been published previously with copyright transfer to another publisher.)

2. Optimization of reaction conditions

Table S1: Optimization of the equivalent of $TMSN_3$.

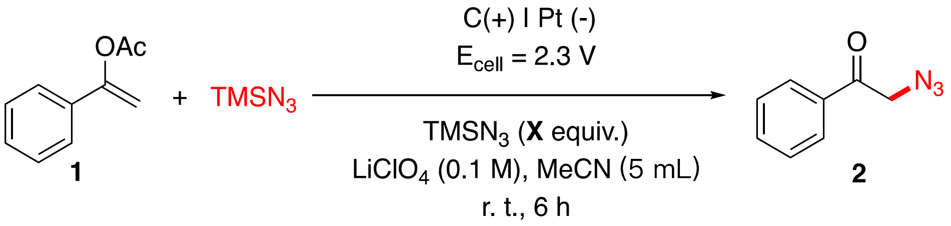

Entry	Variation from the standard reaction conditions	Yield (%)[a]
1	$TMSN_3$ (5 equiv)	23
2[b]	$TMSN_3$ (5 equiv)	14
3	$TMSN_3$ (3 equiv)	41
4	$TMSN_3$ (2 equiv)	**51**
5	$TMSN_3$ (1.5 equiv)	26

[a] isolated yield, [b] constant current = 5 mA

Table S2: Optimization of the equivalent of electrolytes and the reaction time.

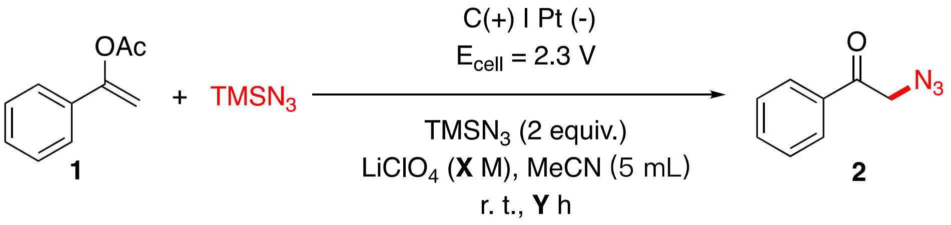

Entry	Variation from the standard reaction conditions	Yield (%)[a]
1	$LiClO_4$ (0.05 M), 6 h	47
2	$LiClO_4$ (0.10 M), 6 h	**51**
3	$LiClO_4$ (0.15 M), 6 h	19
4	$LiClO_4$ (0.20 M), 6 h	22
5	$LiClO_4$ (0.10 M), 4 h	43
6	$LiClO_4$ (0.10 M), 8 h	26

[a] isolated yield

Table S3: Optimization of the electrolytes.

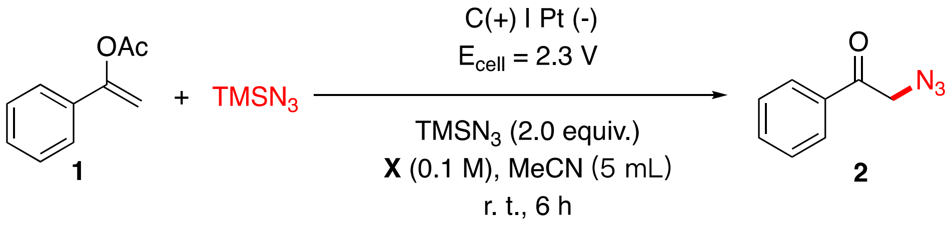

Entry	Variation from the standard reaction conditions	Yield (%)[a]
1	$LiClO_4$	51
2	LiOTf	44
3	Et_4NBF_4	43
4	*n*-Bu_4NBF_4	45
5	*n*-Bu_4NPF_6	**56**
6	*n*-Bu_4NBr	36
7	*n*-Bu_4NI	n. d.
8	*n*-Bu_4NOAc	15
9	*n*-Bu_4NClO_4	50

[a] isolated yield

Table S4: Optimization of the applied cell potentials.

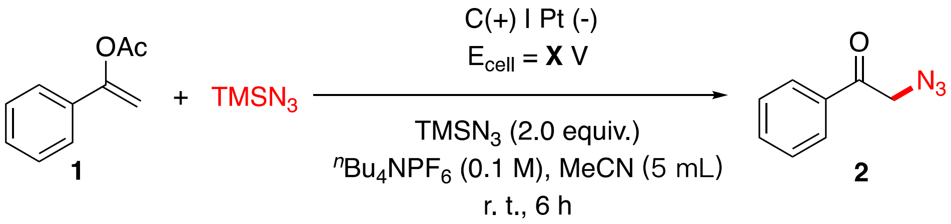

Entry	Variation from the standard reaction conditions	Yield (%)[a]
1	E_{cell} = 2.0 V	48
2	E_{cell} = 2.1 V	50
3	E_{cell} = 2.2 V	55
4	E_{cell} = 2.3 V	**56**
5	E_{cell} = 2.4 V	54
6	E_{cell} = 2.5 V	55

[a] isolated yield

Table S5: Optimization of proton sources.

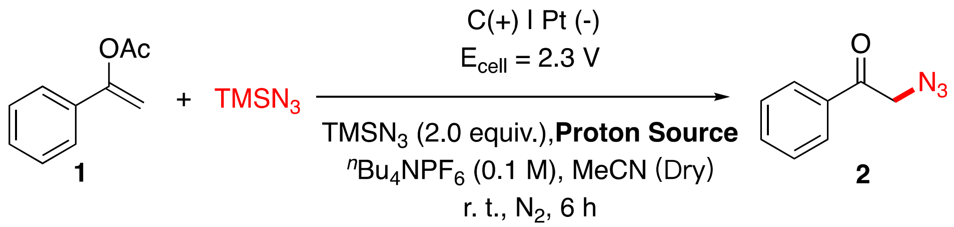

Entry	Proton Source	Yield (%)[a]
1	MeOH (5 equiv)	60
2	*t*-BuOH (5 equiv)	58
3	HFIP (5 equiv)	57
4	H_2O (5 equiv)	**68**
5	H_2O (1.2 equiv)	67
6	H_2O (1 mL)	60

[a] isolated yield

3. General Procedures

General procedure for the preparation of substituted vinyl acetates.

Method A:

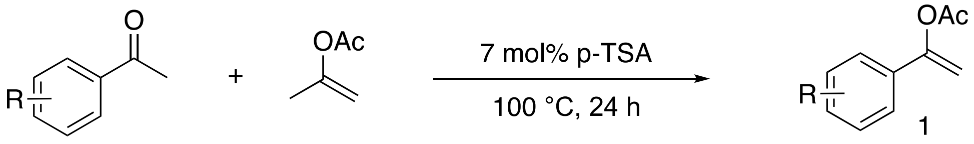

Following the literature procedure[1], to a solution of ketone (10 mmol) in isopropenyl acetate (5.0 mL, 50 mmol) was added *p*-toluenesulfonic acid (120.0 mg, 7 mol %). The reaction mixture was refluxed at 100 ℃ for 24 h, and the remaining isopropenyl acetate was then removed under reduced pressure. The brown oily residue was diluted with ethyl acetate (50 mL), washed with water (15 mL × 3), dried over anhydrous Na_2SO_4, and concentrated under reduced pressure. The crude residue was purified by a silica gel column to give vinyl acetates.

Method B:

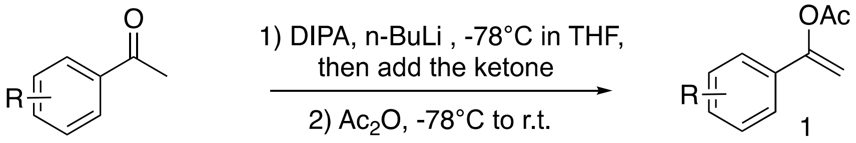

Following the literature procedure[2], *n*-butyllithium (9.6 mmol) was added to a solution of diisopropylamine (9.6 mmol) in THF (40 mL) in a flame-dried round-

bottom flask under an argon atmosphere at −78 °C. The mixture was stirred for 30 min, and then the ketone (8 mmol) was added. The resulting mixture was stirred for 45 min, and then acetic anhydride (16 mmol) was added. The reaction was stirred for 30 min at −78 °C and another 30 min at room temperature. The mixture was poured into saturated $NaHCO_3$ (100 mL) and extracted thrice with EtOAc (60 mL). The combined organic layers were washed with brine and dried over $MgSO_4$, and the solvent was removed under a reduced atmosphere. The crude mixture was purified by column chromatography on silica gel. The spectral data for vinyl acetates are in consistent with those that are available in the literature.

General procedure for the electrochemical synthesis of α-azidoketones

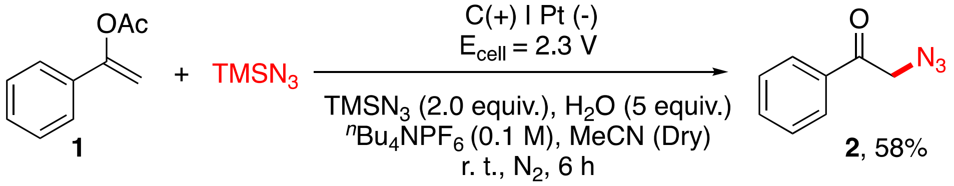

An oven-dried undivided cell as described above was equipped with a stirring bar and $^{n}Bu_4NPF_6$ (1 equiv) was added. The cell was equipped with carbon cloth (15 mm × 15 mm × 0.1 mm) as the anode and platinum plate (15 mm × 15 mm × 0.3 mm) as the cathode. Under the protection of the N_2 atmosphere, anhydrous CH_3CN (5 mL), enol acetate (0.5 mmol), $TMSN_3$ (2 equiv), and H_2O (5 equiv) were injected respectively into the cell via syringes. The reaction mixture was stirred and electrolyzed at a constant cell voltage of 2.3 V at room temperature. Unless noted, the reaction was allowed to react for 6 hours and monitored by TLC. The reaction mixture was concentrated under a reduced atmosphere and the crude mixture was purified by column chromatography on silica gel to yield the desired product.

4. Characterization of Products

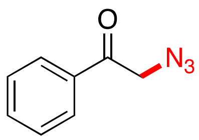

2-Azido-1-phenylethan-1-one (2)[3]

Followed the general procedure, the desired pure product was purified using silica gel chromatography (PE: EA = 20:1) to give 54.4 mg (68% yield) of the product as a yellow oil.

^{1}H NMR (500 MHz, Chloroform-d) δ 7.91 (d, J = 8.1 Hz, 2H), 7.63 (t, J = 7.4 Hz, 1H), 7.50 (t, J = 7.8 Hz, 2H), 4.56 (s, 2H).

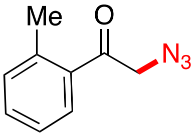

2-Azido-1-(*o*-tolyl)ethan-1-one (3)[3]

Following the general procedure, the desired pure product was purified using silica gel chromatography (PE: EA = 20:1) to give 58.4 mg (67% yield) of the product as a white solid.

^{1}H NMR (400 MHz, Chloroform-d) δ 7.57 (d, J = 7.7 Hz, 1H), 7.44 (t, J = 7.5 Hz, 1H), 7.30 (d, J = 8.3 Hz, 2H), 4.45 (s, 2H), 2.56 (s, 3H).

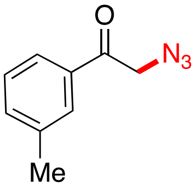

2-Azido-1-(*m*-tolyl)ethan-1-one (4)[3]

Following the general procedure, the desired pure product was purified using silica gel chromatography (PE: EA = 20:1) to give 61.9 mg (71% yield) of the product as a white solid.

^{1}H NMR (500 MHz, Chloroform-d) δ 7.72–7.66 7.72-7.66 (m, 2H), 7.43 (d, J = 7.6 Hz, 1H), 7.36 (d, J = 7.6 Hz, 1H), 4.54 (s, 2H), 2.41 (s, 3H).

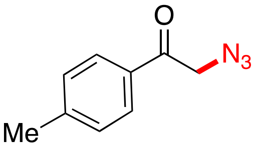

2-Azido-1-(*p*-tolyl)ethan-1-one (5)[3]

Followed the general procedure, the desired pure product was purified using silica gel chromatography (PE: EA = 20:1) to give 65.1 mg (74% yield) of the product as a white solid.

^{1}H NMR (500 MHz, Chloroform-d) δ 7.79 (d, J = 8.3 Hz, 2H), 7.28 (d, J = 7.9 Hz, 2H), 4.52 (s, 2H), 2.42 (s, 3H).

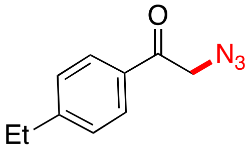

2-Azido-1- (4-ethylphenyl)ethan-1-one (6)

Following the general procedure, the desired pure product was purified using silica gel chromatography (PE: EA = 20:1) to give 71.9 mg (76% yield) of the product as a white solid.

IR (neat, cm^{-1}): 2099 (s), 1691 (m), 1605 (m), 1221 (m), 1182 (m). ^{1}H NMR (400 MHz, Chloroform-d) δ 7.82 (d, *J* = 8.2 Hz, 2H), 7.31 (d, *J* = 8.1 Hz, 2H), 4.53 (s, 2H), 2.71 (q, *J* = 7.6 Hz, 2H), 1.25 (t, *J* = 7.6 Hz, 3H). ^{13}C NMR (101 MHz, Chloroform-d) δ 193.0, 151.5, 132.2, 128.6, 128.3, 54.9, 29.2, 15.2. HRMS (ESI) calculated for $C_{10}H_{12}NO^+$ $[M-N_2]^+$: 162.0913; found: 162.0911.

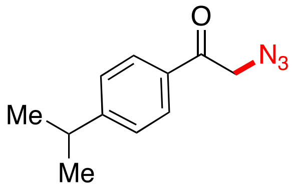

2-Azido-1-(4-isopropylphenyl)ethan-1-one (7)[4]

Following the general procedure, the desired pure product was purified using silica gel chromatography (PE: EA = 20:1) to give 40.1 mg (40% yield) of the product as a white solid.

^{1}H NMR (500 MHz, Chloroform-d) δ 7.87–7.82 7.87-7.82 (m, 2H), 7.34 (d, *J* = 8.3 Hz, 2H), 4.54 (s, 2H), 2.98 (hept, *J* = 6.9 Hz, 1H), 1.27 (d, *J* = 6.9 Hz, 6H)

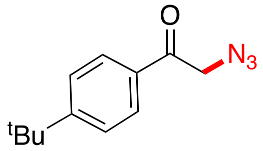

2-Azido-1-(4-(*tert*-butyl)phenyl)ethan-1-one (8)[3]

Following the general procedure, the desired pure product was purified using silica gel chromatography (PE: EA = 20:1) to give 82.9 mg (76% yield) of product as a white solid.

^{1}H NMR (400 MHz, Chloroform-d) δ 7.84 (d, *J* = 8.4 Hz, 2H), 7.50 (d, *J* = 8.4 Hz, 2H), 4.53 (s, 2H), 1.34 (s, 9H)

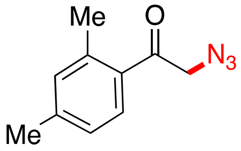

2-Azido-1-(2,4-dimethylphenyl)ethan-1-one (9)

Following the general procedure (in 0.3 mmol scale), the desired pure product was purified using silica gel chromatography (PE: EA = 20:1) to give 40.3 mg (71% yield) of the product as a white solid.

IR (neat, cm^{-1}): 2104 (s), 1691 (m), 1612 (w), 1281 (w), 1214 (m). ^{1}H NMR (500 MHz, Chloroform-d) δ 7.49 (d, *J* = 7.9 Hz, 1H), 7.13 – 7.06 7.13-7.06 (m, 2H), 4.44 (s, 2H), 2.54 (s, 3H), 2.37 (s, 3H). ^{13}C NMR (126 MHz, Chloroform-d) δ 195.6, 143.6, 140.2, 133.6, 131.5, 129.0, 126.7, 56.4, 21.9, 21.6. HRMS (ESI) calculated for $C_{10}H_{12}NO^+$ $[M-N_2]^+$: 162.0913; found: 162.0912.

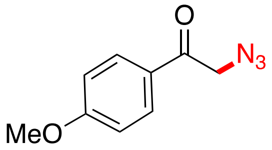

2-Azido-1-(4-methoxyphenyl)ethan-1-one (10)[3]

Following the general procedure, the desired pure product was purified using silica gel chromatography (PE: EA = 10:1) to give 59.4 mg (62% yield) of the product as a white solid.

^{1}H NMR (400 MHz, Chloroform-d) δ 7.86 (d, *J* = 8.8 Hz, 2H), 6.94 (d, *J* = 8.8 Hz, 2H), 4.48 (s, 2H), 3.86 (s, 3H).

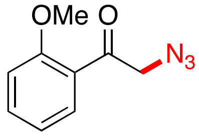

2-Azido-1-(2-methoxyphenyl)ethan-1-one (11)[5]

Following the general procedure, the desired pure product was purified using silica gel chromatography (PE: EA = 10:1) to give 62.9 mg (66% yield) of the product as a white solid.

^{1}H NMR (500 MHz, Chloroform-d) δ 7.91 (dd, *J* = 7.8, 1.8 Hz, 1H), 7.53 (ddd, *J* = 8.5, 7.3, 1.8 Hz, 1H), 7.06 – 7.02 7.06-7.02 (m, 1H), 6.99 (d, *J* = 8.4 Hz, 1H), 4.50 (s, 2H), 3.93 (s, 3H).

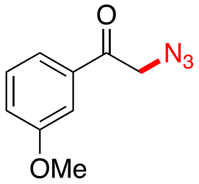

2-Azido-1-(3-methoxyphenyl)ethan-1-one (12)[5]

Following the general procedure, the desired pure product was purified using silica gel chromatography (PE: EA = 10:1) to give 52.6 mg (55% yield) of the product as a white solid.

^{1}H NMR (500 MHz, Chloroform-d) δ 7.46 – 7.43 7.46-7.43 (m, 2H), 7.41 – 7.37 7.41-7.37 (m, 1H), 7.16 (ddd, *J* = 8.0, 2.6, 1.2 Hz, 1H), 4.55 (s, 2H), 3.86 (s, 3H).

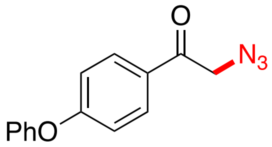

2-Azido-1-(4-phenoxyphenyl)ethan-1-one (13)

Following the general procedure, the desired pure product was purified using silica gel chromatography (PE: EA = 10:1) to give 104.1 mg (82% yield) of the product as a white solid.

IR (neat, cm^{-1}): 2100 (s), 1584 (m), 1488 (m), 1219 (s), 1165 (m). ^{1}H NMR (500 MHz, Chloroform-d) δ 7.90 – 7.85 (m, 2H), 7.40 (tt, J = 7.6, 2.2 Hz, 2H), 7.24 – 7.20 (m, 1H), 7.09 – 7.05 (m, 2H), 7.03 – 6.99 (m, 2H), 4.50 (s, 2H). ^{13}C NMR (101 MHz, Chloroform-d) δ 191.8, 163.0, 155.2, 130.4, 130.3, 129.0, 125.1, 120.5, 117.6, 54.8. HRMS (ESI) calculated for $C_{14}H_{12}NO_2^+$ [M-N_2] $^+$: 226.0863; found: 226.0859.

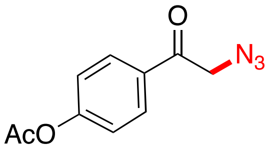

4-(2-Azidoacetyl)phenyl acetate (14)[3]

Following the general procedure, the desired pure product was purified using silica gel chromatography (PE: EA = 10:1) to give 72 mg (66% yield) of the product as a white solid.

^{1}H NMR (400 MHz, Chloroform-d) δ 7.94 (d, J = 8.7 Hz, 2H), 7.23 (d, J = 8.7 Hz, 2H), 4.54 (s, 2H), 2.33 (s, 3H).

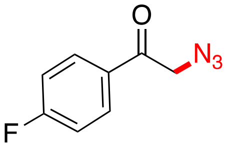

2-Azido-1-(4-fluorophenyl)ethan-1-one (15)[3]

Following the general procedure, the desired pure product was purified using silica gel chromatography (PE: EA = 20:1) to give 39.1 mg (44% yield) of the product as a yellow oil.

^{1}H NMR (400 MHz, Chloroform-d) δ 7.95 (dd, J = 7.8, 5.5 Hz, 2H), 7.18 (t, J = 8.2 Hz, 2H), 4.53 (s, 2H). ^{19}F NMR (471 MHz, $CDCl_3$) δ -104.2 (m).

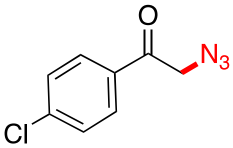

2-Azido-1-(4-chlorophenyl)ethan-1-one (16)[3]

Following the general procedure, the desired pure product was purified using silica gel chromatography (PE: EA = 20:1) to give 47.1 mg (48% yield) of the product as a yellow oil.

^{1}H NMR (400 MHz, Chloroform-d) δ 7.85 (d, J = 7.9 Hz, 2H), 7.47 (d, J = 7.9 Hz, 2H), 4.53 (s, 2H).

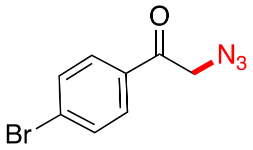

2-Azido-1-(4-bromophenyl)ethan-1-one (17)[3]

Followed the general procedure, the desired pure product was purified using silica gel chromatography (PE: EA = 20:1) to give 35.3 mg (30% yield) of the product as a yellow solid.

^{1}H NMR (400 MHz, Chloroform-d) δ 7.77 (d, J = 7.6 Hz, 2H), 7.65 (d, J = 7.6 Hz, 2H), 4.53 (s, 2H).

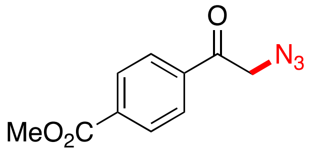

Methyl 4-(2-azidoacetyl)benzoate (18)[6]

Following the general procedure, the desired pure product was purified using silica gel chromatography (PE: EA = 10:1) to give 50.2 mg (46% yield) of the product as a white solid.

^{1}H NMR (400 MHz, Chloroform-d) δ 8.15 (d, J = 8.6 Hz, 2H), 7.96 (d, J = 8.6 Hz, 2H), 4.59 (s, 2H), 3.96 (s, 3H).

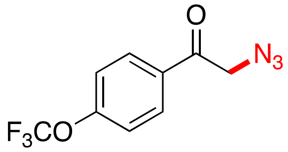

2-Azido-1-(4-(trifluoromethoxy)phenyl)ethan-1-one (19)[7]

Following the general procedure, the desired pure product was purified using silica gel chromatography (PE: EA = 20:1) to give 47.3 mg (39% yield) of the product as a yellow oil.

^{1}H NMR (400 MHz, Chloroform-d) δ 7.97 (d, J = 8.7 Hz, 2H), 7.33 (d, J = 8.5 Hz, 2H), 4.54 (s, 2H). ^{19}F NMR (471 MHz, Chloroform-d) δ -57.5 (m).

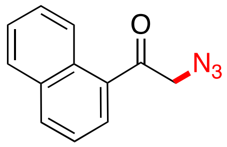

2-Azido-1-(naphthalen-1-yl)ethan-1-one (20)[6]

Following the general procedure, the desired pure product was purified using silica gel chromatography (PE: EA = 20:1) to give 54.1 mg (51% yield) of the product as a white solid.

^{1}H NMR (400 MHz, Chloroform-d) δ 8.74 (d, J = 8.6 Hz, 1H), 8.05 (d, J = 8.2 Hz, 1H), 7.89 (d, J = 8.1 Hz, 1H), 7.80 (d, J = 7.2 Hz, 1H), 7.66 – 7.61 (m, 1H), 7.57 (t, J = 7.5 Hz, 1H), 7.50 (t, J = 7.7 Hz, 1H), 4.57 (s, 2H).

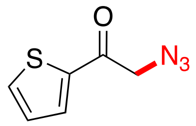

2-Azido-1-(thiophen-2-yl)ethan-1-one (21) [4]

Following the general procedure, the desired pure product was purified using silica gel chromatography (PE: EA = 20:1) to give 61.5 mg (74% yield) of the product as a white solid.

^{1}H NMR (400 MHz, Chloroform-d) δ 7.74–7.70 (m, 2H), 7.16 (dd, *J* = 4.8, 4.0 Hz, 1H), 4.45 (s, 2H).

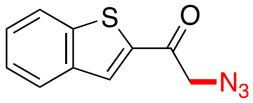

2-Azido-1-(benzo[*b*]thiophen-2-yl)ethan-1-one (22)[8]

Following the general procedure (in 0.3 mmol scale), the desired pure product was purified using silica gel chromatography (PE: EA = 20:1) to give 38.1 mg (58% yield) of the product as a white solid.

^{1}H NMR (500 MHz, Chloroform-d) δ 7.96 (s, 1 H), 7.93–7.87 (m, 2H), 7.51 (ddd, *J* = 8.2, 7.1, 1.3 Hz, 1H), 7.44 (ddd, *J* = 8.1, 7.2, 1.1 Hz, 1H), 4.56 (s, 2H). ^{13}C NMR (126 MHz, Chloroform-d) δ 188.0, 142.7, 140.2, 138.9, 129.9, 128.2, 126.3, 125.5, 123.2, 55.0.

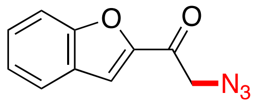

2-Azido-1-(benzofuran-2-yl)ethan-1-one (23)[9]

Following the general procedure (in 0.3 mmol scale), the desired pure product was purified using silica gel chromatography (PE: EA = 20:1) to give 31.2 mg (52% yield) of the product as a white solid.

^{1}H NMR (500 MHz, Chloroform-d) δ 7.74 (dt, *J* = 7.9, 0.9 Hz, 1H), 7.62 (d, *J* = 0.9 Hz, 1H), 7.60 – 7.56 (m, 1H), 7.52 (ddd, *J* = 8.4, 7.1, 1.2 Hz, 1H), 7.35 (ddd, *J* = 8.0, 7.0, 1.0 Hz, 1H), 4.56 (s, 2H).

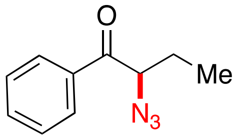

2-azido-1-phenylbutan-1-one (24)[10]

Following the general procedure, the desired pure product was purified using silica gel chromatography (PE: EA = 20:1) to give 82.1 mg (87% yield) of the product as a colorless oil.

^{1}H NMR (400 MHz, Chloroform-d) δ 7.96–7.89 (m, 2H), 7.61 (t, *J* = 7.4 Hz, 1H), 7.49 (t, *J* = 7.7 Hz, 2H), 4.54 (dd, *J* = 8.5, 5.0 Hz, 1H), 2.04–1.93 (m, 1H), 1.90-1.81 (m, 1H), 1.07 (t, *J* = 7.4 Hz, 3H).

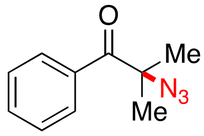

2-Azido-2-methyl-1-phenylpropan-1-one (25)[11]
Following the general procedure, the desired pure product was purified using silica gel chromatography (PE: EA = 20:1) to give 61.6 mg (60% yield) of the product as a colorless oil.
^{1}H NMR (400 MHz, Chloroform-d) δ 8.13 – 8.07 (m, 2H), 7.56 (tt, *J* = 6.8, 1.2 Hz, 1H), 7.46 (t, *J* = 7.6 Hz, 2H), 1.62 (s, 6H).

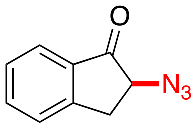

2-Azido-2,3-dihydro-1*H*-inden-1-one (26) [12]
Following the general procedure (reaction time = 2 h), the desired pure product was purified using silica gel chromatography (PE: EA = 20:1) to give 13.8 mg (22% yield) of the product as a yellow solid.
^{1}H NMR (500 MHz, Chloroform-d) δ 7.80 (d, *J* = 7.6 Hz, 1H), 7.65 (t, *J* = 7.5 Hz, 1H), 7.48 – 7.40 (m, 2H), 4.32 (dd, *J* = 8.1, 4.6 Hz, 1H), 3.51 (dd, *J* = 17.1, 8.1 Hz, 1H), 2.94 (dd, *J* = 17.1, 4.6 Hz, 1H).

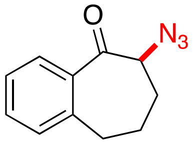

6-Azido-6,7,8,9-tetrahydro-5*H*-benzo[7]annulen-5-one (27)[13]
Following the general procedure, the desired pure product was purified using silica gel chromatography (PE: EA = 20:1) to give 61.5 mg (49% yield) of the product as a white solid.
^{1}H NMR (400 MHz, Chloroform-d) δ 7.78 (dd, *J* = 7.7, 1.1 Hz, 1H), 7.44 (td, *J* = 7.5, 1.4 Hz, 1H), 7.33 (t, *J* = 7.4 Hz, 1H), 7.23 (d, *J* = 7.6 Hz, 1H), 4.27 (dd, *J* = 10.7, 5.3 Hz, 1H), 3.03 – 2.94 (m, 2H), 2.24 – 2.10 (m, 2H), 2.00 – 1.62 (m, 1H), 1.85 – 1.76 (m, 1H).

5. Unsuccessful Substrates

Unfortunately, the current protocol was not applicable to the oxyazidolation of enol acetate deriving from aliphatic ketones.

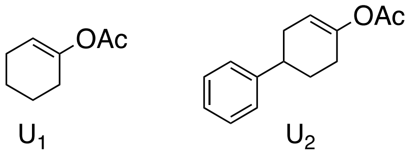

6. Derivatizations of the product

Procedure for the click reaction[7]

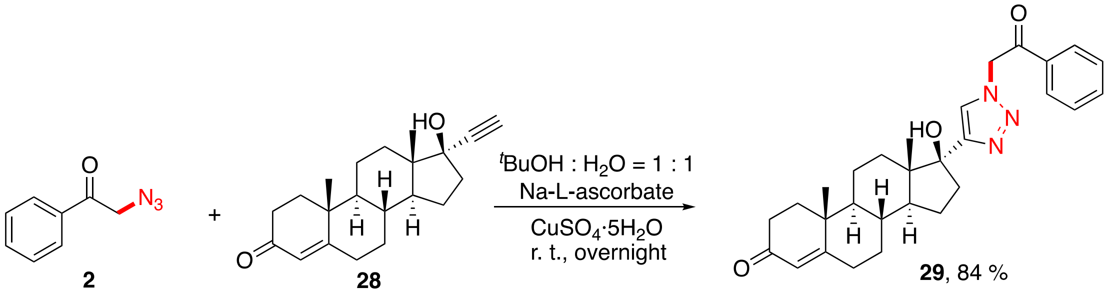

A solution of 2-azido-1-phenylethan-1-one (**2**) (40.3 mg, 0.25 mmol) and ethisterone **28** (78.1 mg, 0.25 mmol) in presence of $CuSO_4 \cdot 5H_2O$ (125 mg, 0.5 mmol) and sodium ascorbate (98 mg, 0.5 mmol) in 5 mL H_2O/*t*-BuOH (1:1) was stirred at room temperature under an argon atmosphere for 12 h followed purification by column chromatography on silica gel (10:1 v/v DCM/MeOH) to give 99.3 mg of product **29** as a white solid (86% yield).

IR (neat, cm^{-1}): 2939 (s), 1702 (s), 1662 (s), 1228 (s), 690 (m). ^1H NMR (400 MHz, Chloroform-d) δ 7.95 (d, *J* = 7.8 Hz, 2H), 7.63 (t, *J* = 7.4 Hz, 1H), 7.54 – 7.48 (m, 3H), 5.82 (d, *J* = 2.3 Hz, 2H), 5.66 (s, 1H), 3.09 (s, 1H), 2.42 – 2.21 (m, 5H), 2.15 – 2.06 (m, 1H), 1.95 – 1.80 (m, 3H), 1.65 – 1.29 (m, 7H), 1.15 (s, 3H), 1.05 (s, 4H), 0.75 – 0.68 (m, 1H), 0.55 – 0.48 (m, 1H). ^{13}C NMR (101 MHz, Chloroform-d) δ 199.9, 190.7, 171.7, 154.3, 134.9, 134.2, 129.4, 128.4, 124.0, 123.5, 82.4, 55.8, 53.5, 49.1, 47.2, 38.9, 38.1, 36.5, 35.9, 34.2, 33.1, 32.9, 31.9, 24.0, 20.9, 17.7, 14.6. HRMS (ESI) calculated for $C_{29}H_{36}N_3O_3^+$ [M + H] $^+$: 474.2751; found: 474.2748.

Procedure for the synthesis of 3-substituted isoquinoline[5]

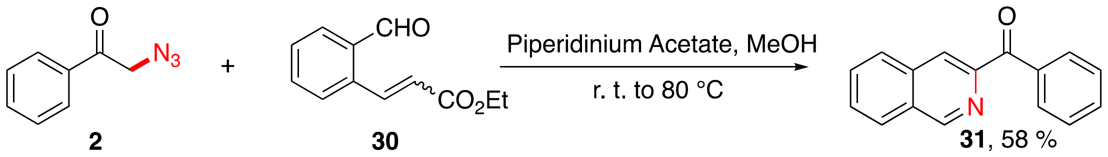

2-(Formylphenyl)acrylate **30** (102.1 mg, 0.5 mmol), α-azidoketone (80.6 mg, 0.5 mmol), and MeOH (3.0 mL) were taken in a 10 mL round bottom flask, and then freshly prepared piperidinium acetate (145.2 mg, 1.0 mmol) was added. The reaction mixture was initially stirred at room temperature for 3 h, and after that, the reaction temperature was increased to 80 °C in the oil bath and stirred at this temperature for another 3 h. After complete consumption of starting materials, (reaction monitored by TLC), MeOH was removed under reduced pressure and a crude product was obtained which was purified by column chromatography using a mixture of ethyl acetate/petroleum ether (40 %) on silica gel to provide the desired product (**31**) in a 58% isolated yield.

^{1}H NMR (500 MHz, Chloroform-d) δ 9.34 (s, 1H), 8.47 (s, 1H), 8.08 (dd, J = 8.3, 1.3 Hz, 3H), 8.01 (d, J = 8.1 Hz, 1H), 7.83 – 7.74 (m, 2H), 7.63 – 7.59 (m, 1H), 7.53 – 7.49 (m, 2H).

7. Mechanism Studies

7.1 Cyclic Voltammetry Studies

General information: Cyclic voltammetry (CV) experiments were conducted in a 10 mL glass vial fitted with a glassy carbon working electrode (3 mm in diameter), a platinum wire auxiliary electrode and SCE reference electrode. The scan rate was 0.1 V/s. All the data was obtained in the background of 0.1 M n-Bu_4NPF_6. Current was reported in mA, while all potentials were reported in V against the $Fc^{+/0}$ redox couple.

The cyclic voltammetry studies show while there is no obvious oxidation peak for $TMSN_3$,[14] 1-phenylvinylacetate (**1**) exhibits two oxidation peaks. The first peak ($E_{p/2}$ = 1.51 V vs $Fc^{+/0}$) was assigned to be the oxidation of the vinyl acetate moiety.

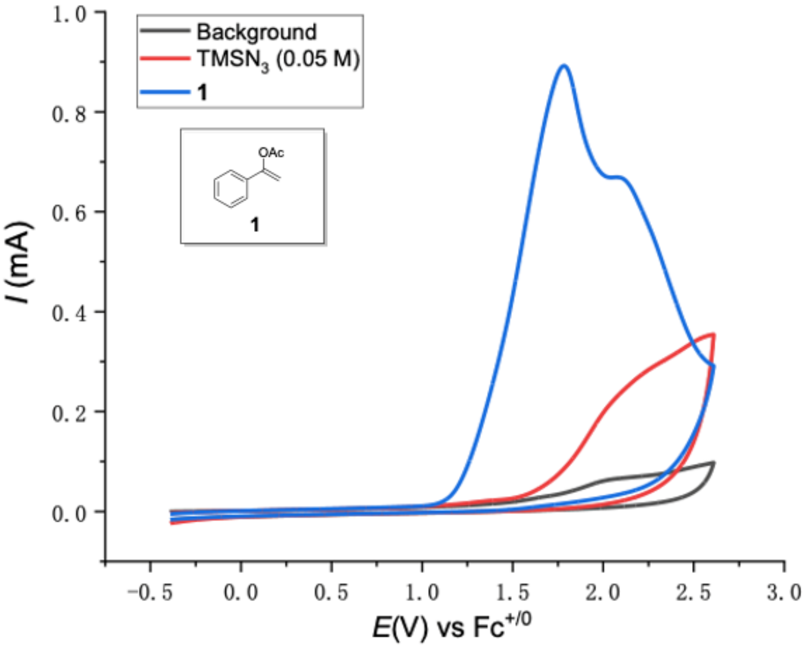

In the electrolysis of the isopropyl-substituted substrate, the starting material **7a** was fully consumed. Unfortunately, attempts to identify the byproduct(s) were unsuccessful. The cyclic voltammetry studies show a similar redox profile for the oxidation of vinylacetate moiety ($E_{p/2}$ = 1.52 V vs $Fc^{+/0}$) of compound **7a**. In addition, the oxidation of isopropylbenzene (**7b**) might constitute the possible side-reaction as proved by its low onset oxidation potential (E_{onset} = 1.68 V vs $Fc^{+/0}$), which has also been previously documented in the literature （*Nature*, **2015**, 517, 600-604; *J. Am. Chem. Soc.* **2020**, *142*, 41, 17693–17702).

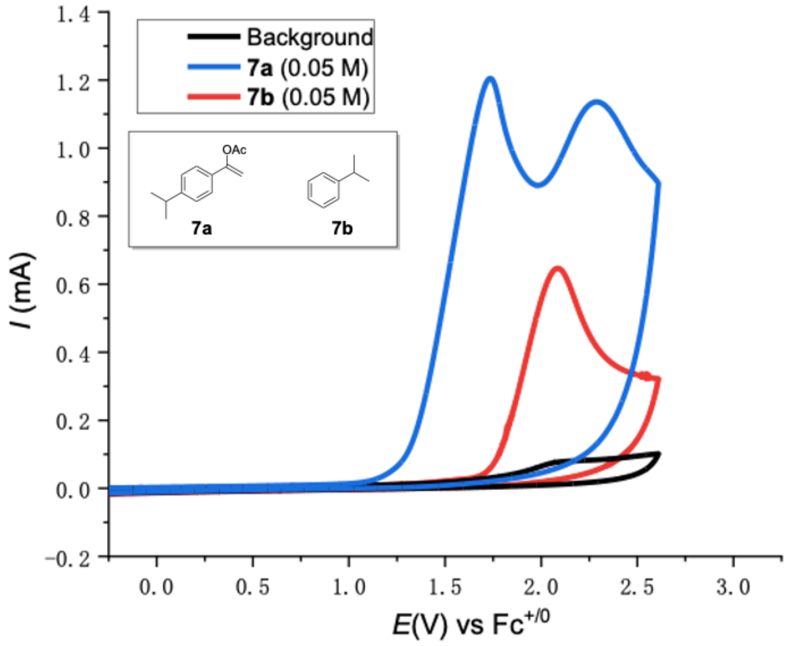

7.2 ^{18}O Labelling Experiment

We have conducted a $H_2{}^{18}O$ experiment and found there was no ^{18}O incorporation in the obtained α-azidoketone (**2**). Therefore, the oxygen source of the newly formed carbonyl moiety should originate directly from the vinyl acetate.

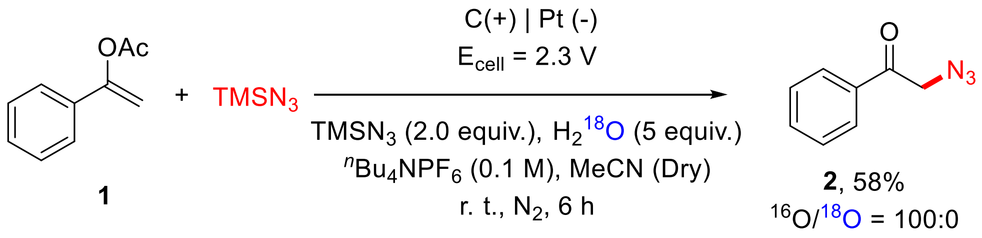

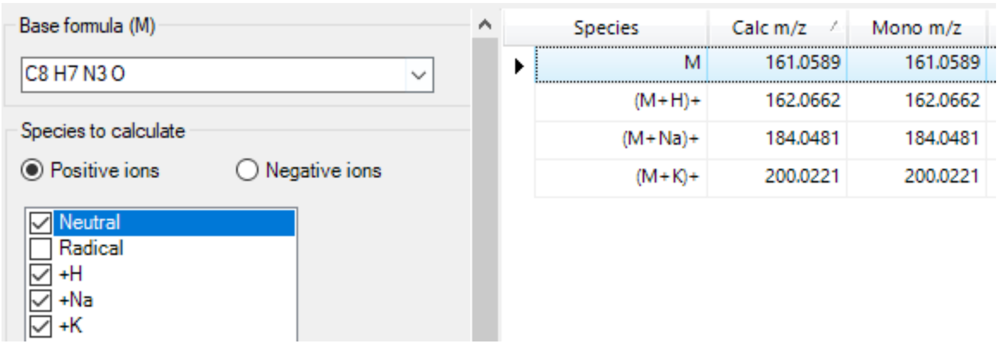

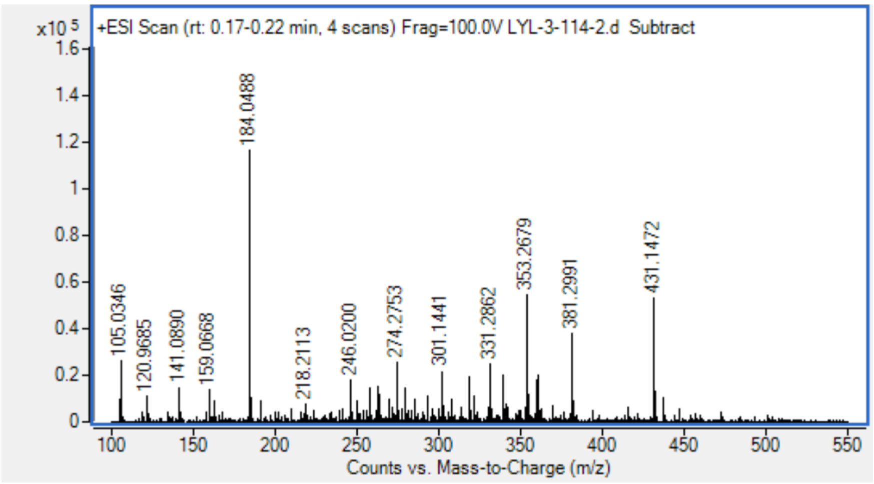

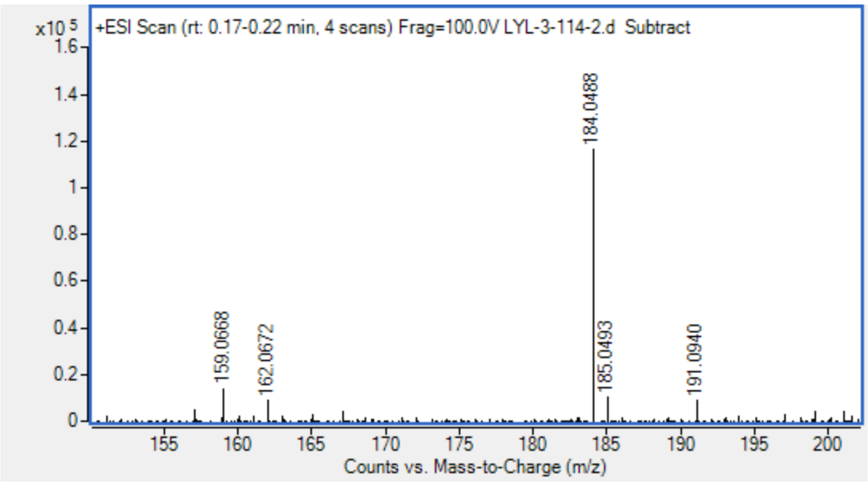
x10 5
+ESI Scan (rt: 0.17-0.22 min, 4 scans) Frag=100.0V LYL-3-114-2.d Subtract
1.6
1.4
1.2
1
0.8
0.6
0.4
0.2
0
159.0668
162.0672
184.0488
185.0493
191.0940
155
160
165
170
175
180
185
190
195
200
Counts vs. Mass-to-Charge (m/z)

8. References

1. He, R.-D.; Li, C.-L.; Pan, Q.-Q.; Guo, P.; Liu, X.-Y.; Shu, X.-Z. *J. Am. Chem. Soc.* **2019**, *141*, 12481-12486.
2. Basdevant, B.; Legault, C. Y. *Org. Lett.* **2015**, *17*, 4918-4921.
3. Wei, W.; Cui, H.; Yue, H.; Yang, D. *Green Chem.* **2018**, *20*, 3197-3202.
4. Zheng, Z.-J.; Yu, T.-Y.; Xu, P.-F.; Wei, H. *Asian J. Org. Chem.* **2018**, *7*, 1579-1582.
5. Prasad, B.; Phanindrudu, M.; Tiwari, D. K.; Kamal, A. *J. Org. Chem.* **2019**, *84*, 12334-12343.
6. Chandra, A.; Parida, K. N.; Moorthy, J. N. *Tetrahedron* **2017**, *73*, 5827-5832.
7. Bangalore, P. K.; Vagolu, S. K.; Bollikanda, R. K.; Veeragoni, D. K.; Choudante, P. C.; Misra, S.; Sriram, D.; Sridhar, B.; Kantevari, S. *J. Nat. Prod.* **2020**, *83*, 26-35.
8. Murar, M.; Dobias, J.; Sramel, P.; Addova, G.; Hanquet, G.; Bohac, A. *Eur. J. Med Chem.* **2017**, *126*, 754-761.
9. Hossain, A.; Vidyasagar, A.; Eichinger, C.; Lankes, C.; Phan, J.; Rehbein, J.; Reiser, O. *Angew. Chem. Int. Ed. Engl.* **2018**, *57*, 8288-8292.
10. Zhang, Z.; Luo, Y.; Du, H.; Xu, J.; Li, P. *Chem. Sci.* **2019**, *10*, 5156-5161.
11. Uyanik, M.; Sahara, N.; Tsukahara, M.; Hattori, Y.; Ishihara, K. *Angew. Chem. Int. Ed. Engl.* **2020**, *59*, 17110-17117.
12. Siu, J. C.; Sauer, G. S.; Saha, A.; Macey, R. L.; Fu, N.; Chauviré, T.; Lancaster, K. M.; Lin, S. *J. Am. Chem. Soc.* **2018**, *140*, 12511-12520.
13. Eva, J. T., Tamas, P. *Eur. J. Org. Chem.* **2002**, *17*, 3055-3064.
14. Ye, Z.; Zhu, R.; Wang, F.; Jiang, H.; Zhang, F. *Org. Lett.* **2021**, *23*, 8240-8245.

9. Spectral Data (^{1}H, ^{13}C, ^{19}F) of Products

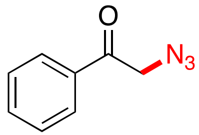

2 ^{1}H NMR (500 MHz, $CDCl_3$)

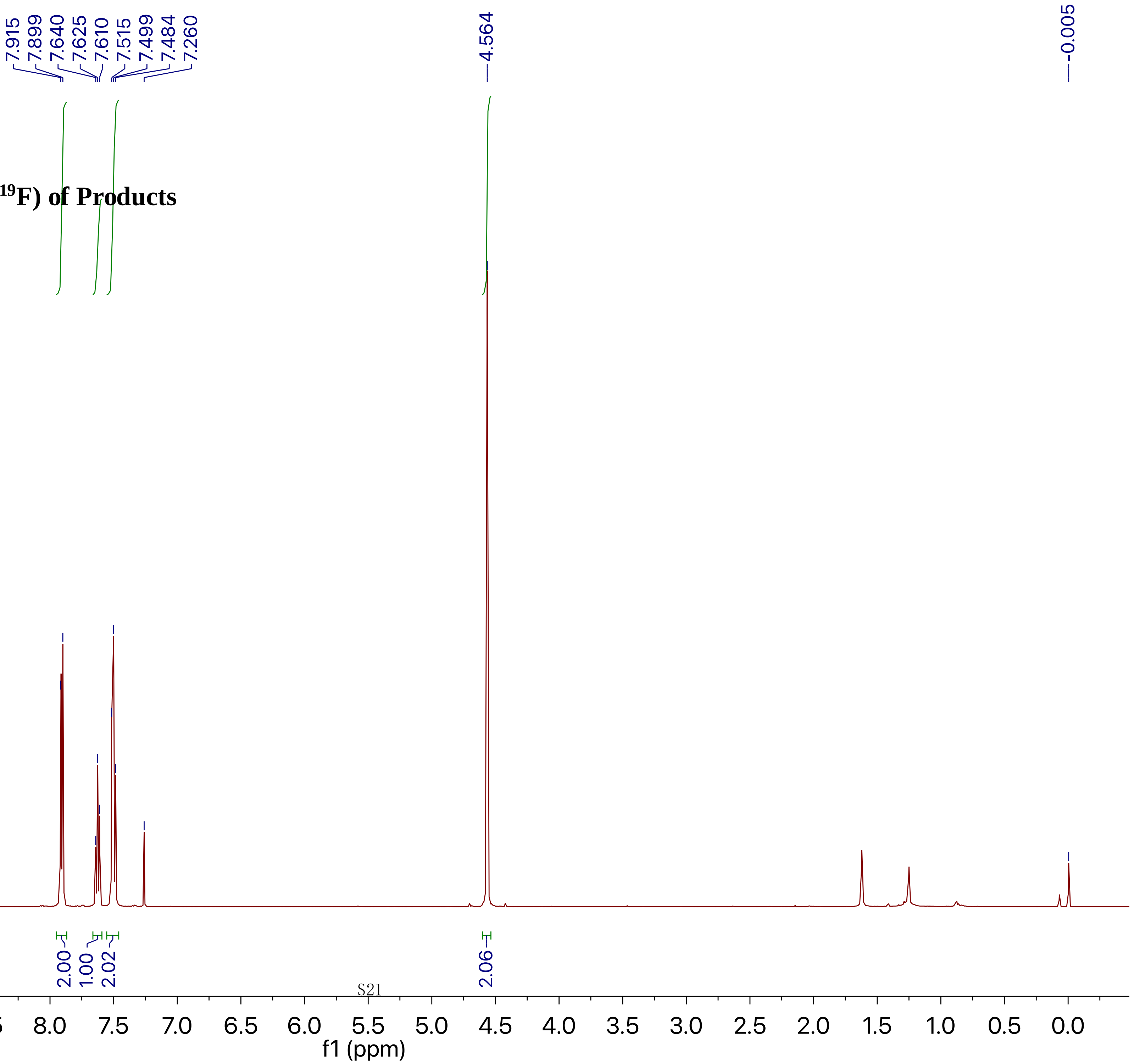

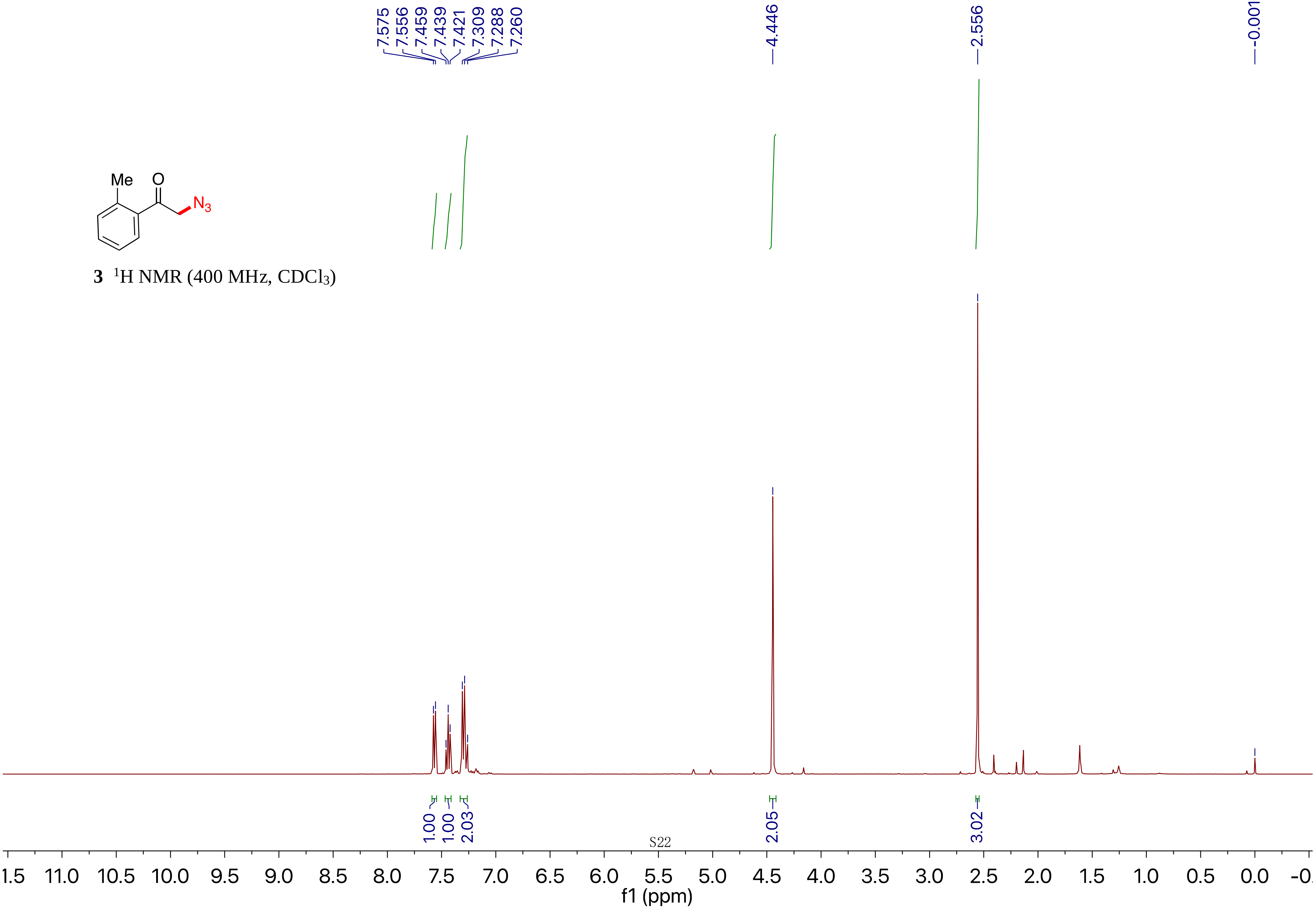

3 ^{1}H NMR (400 MHz, $CDCl_3$)

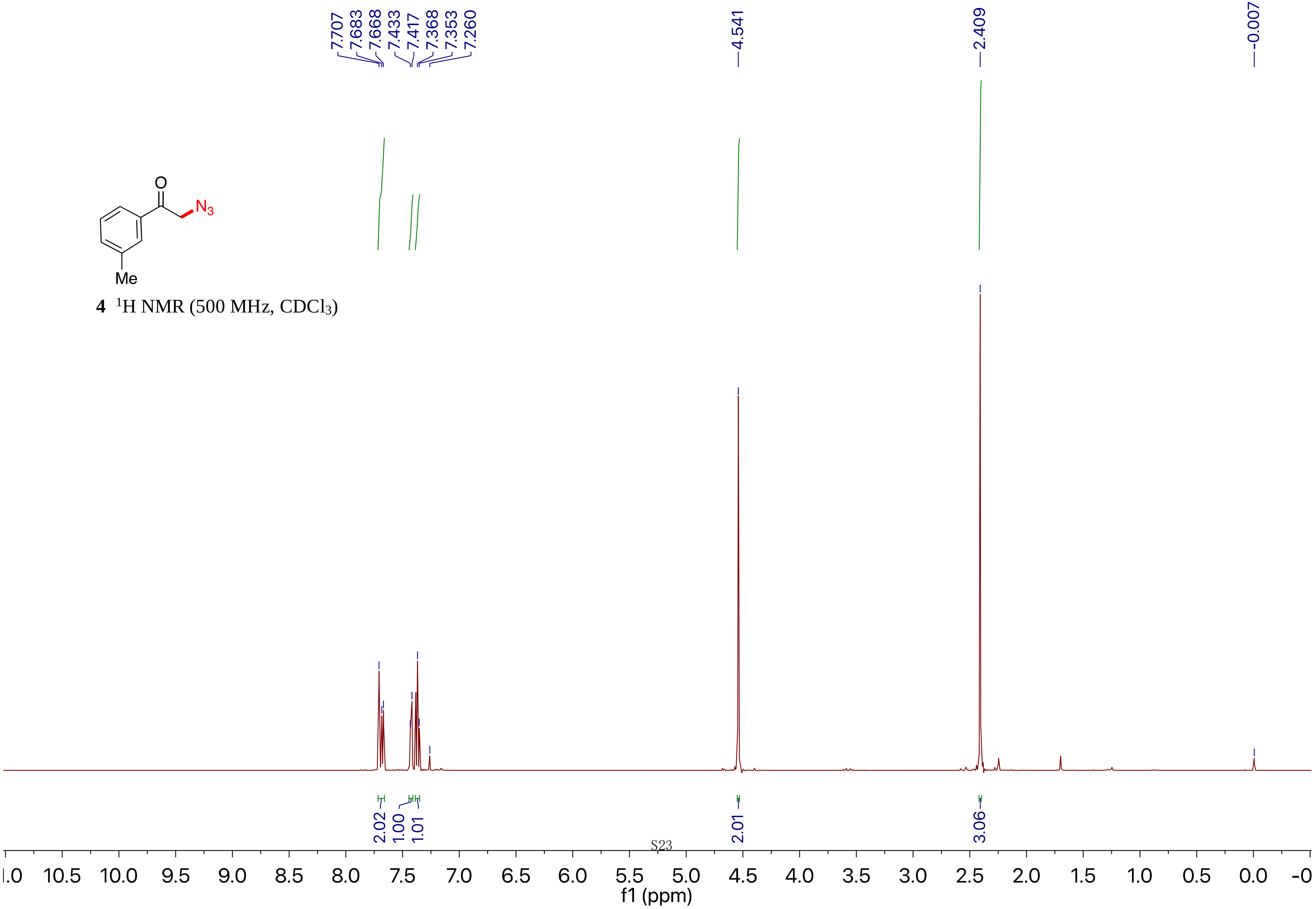

4 ^{1}H NMR (500 MHz, $CDCl_3$)

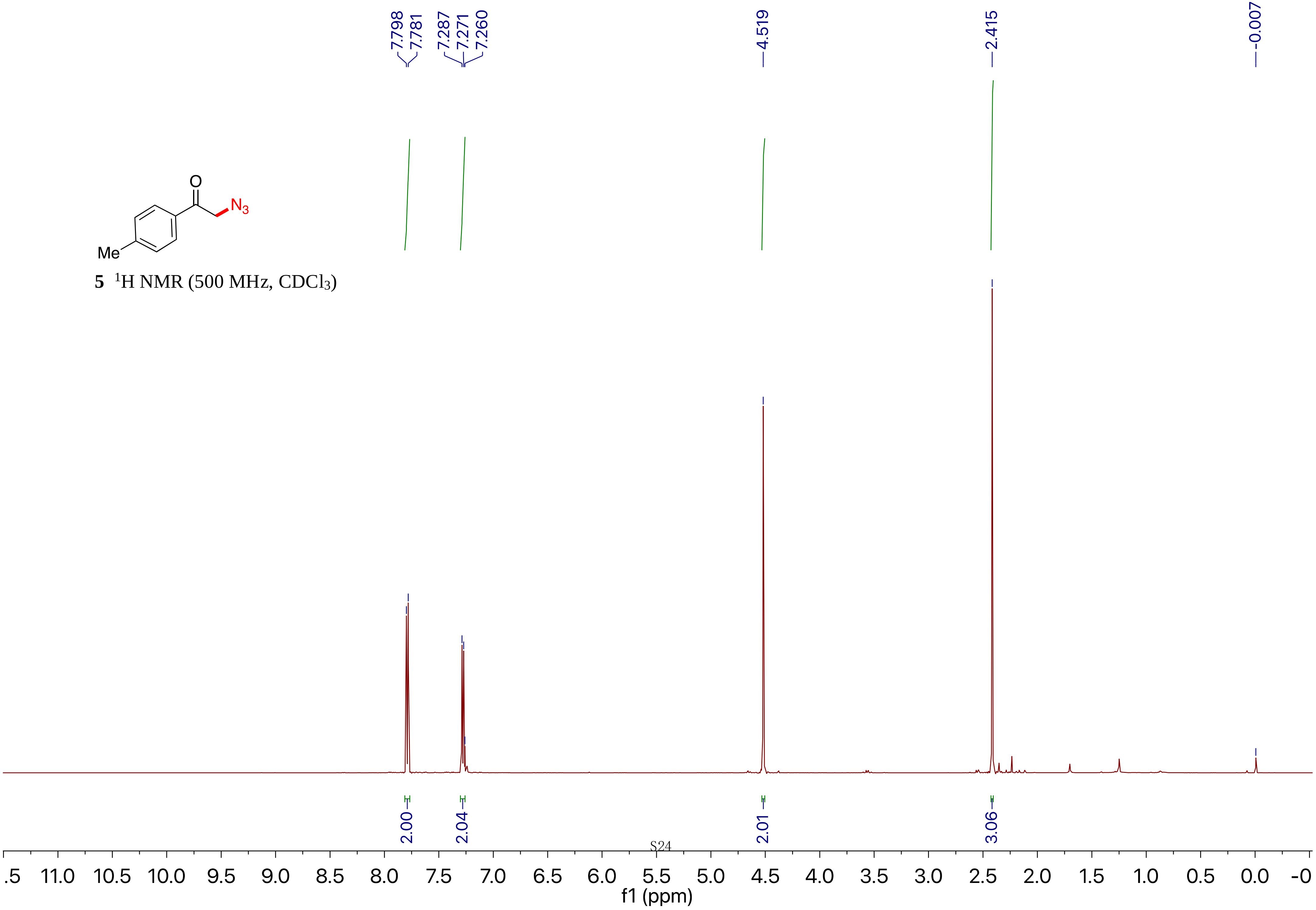

5 ^{1}H NMR (500 MHz, $CDCl_3$)

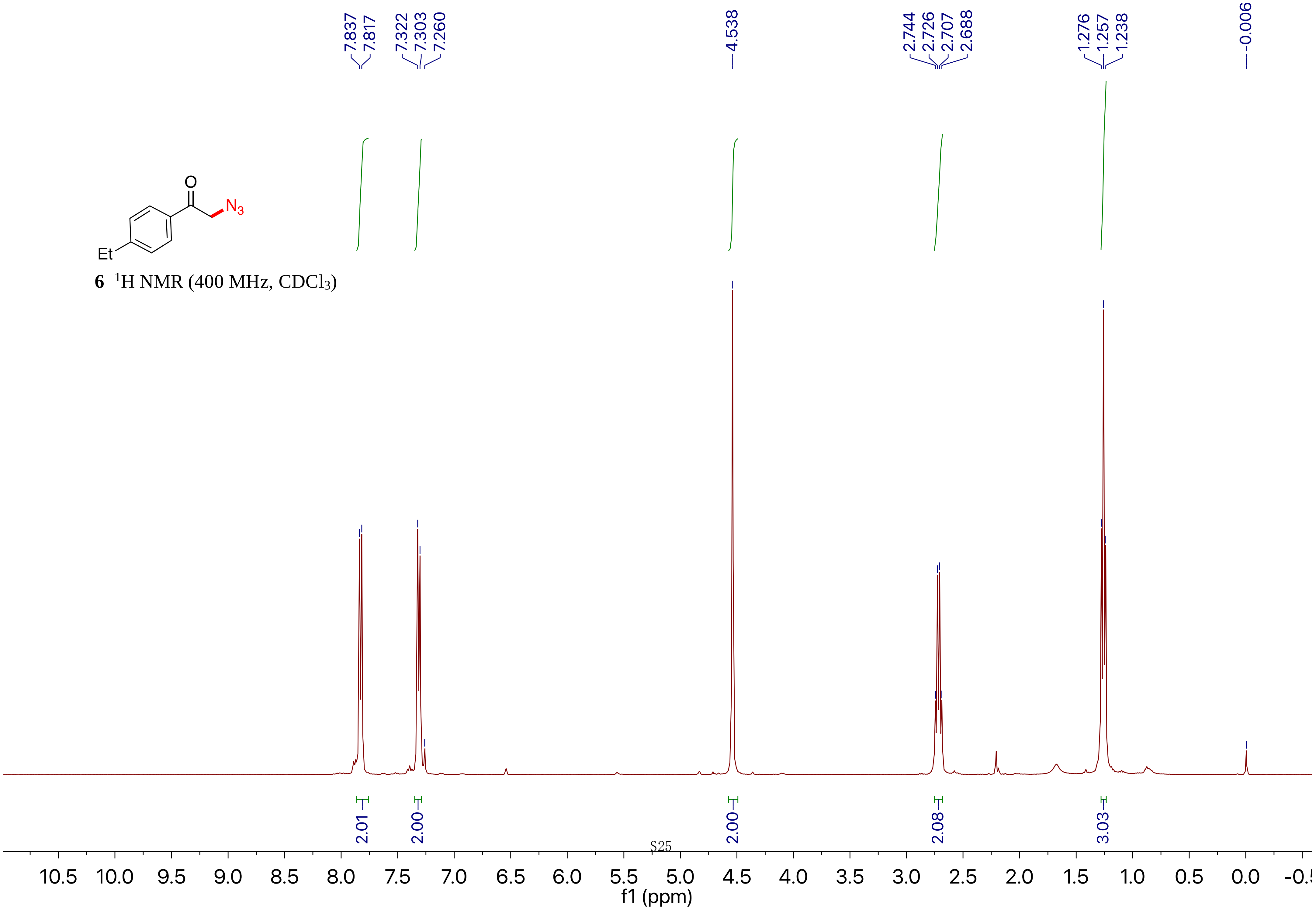

6 ^{1}H NMR (400 MHz, $CDCl_3$)

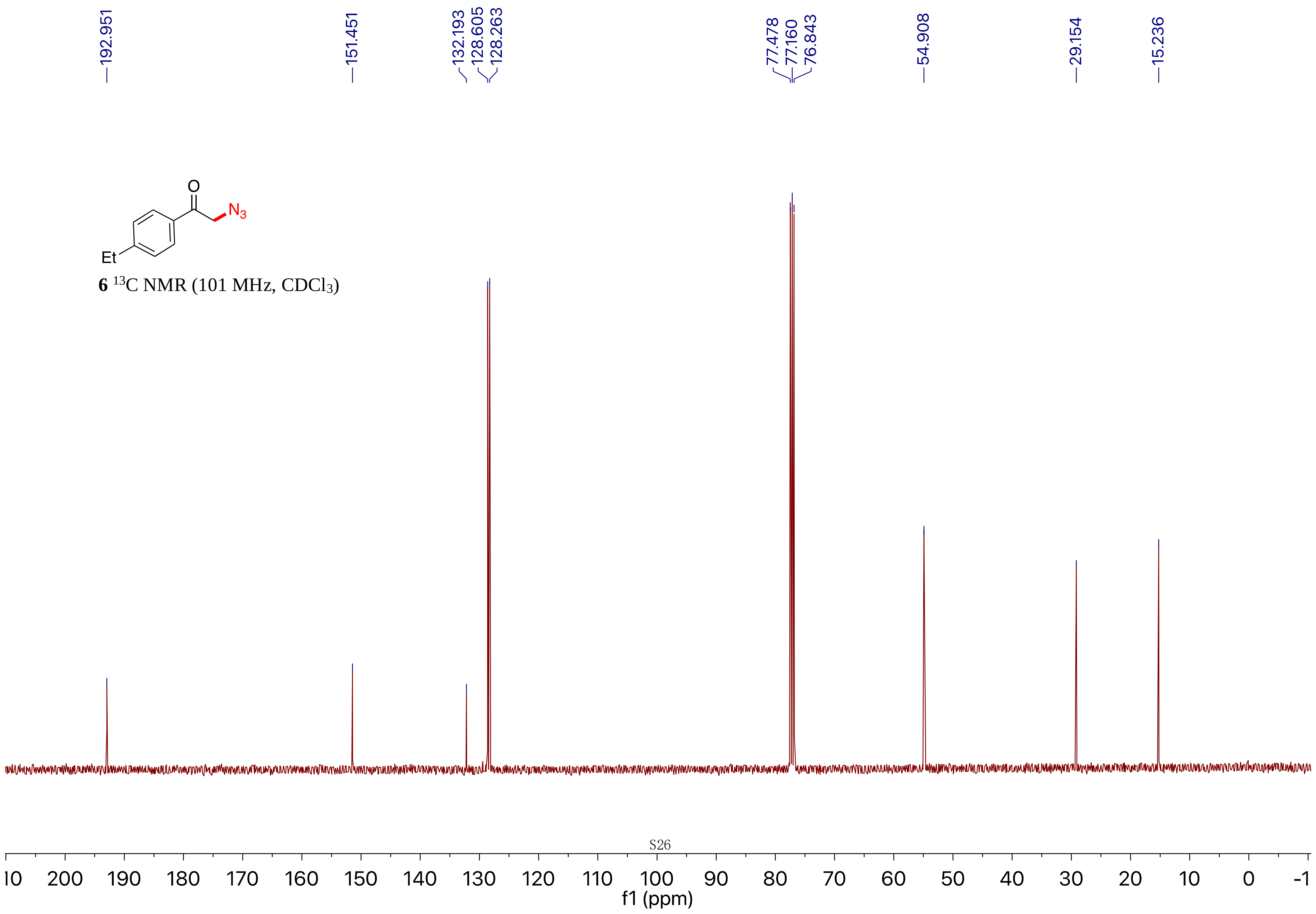
192.951
151.451
132.193
128.605
128.263
77.478
77.160
76.843
54.908
29.154
15.236
O
N3
Et
6 13C NMR (101 MHz, CDCl3)
f1 (ppm)

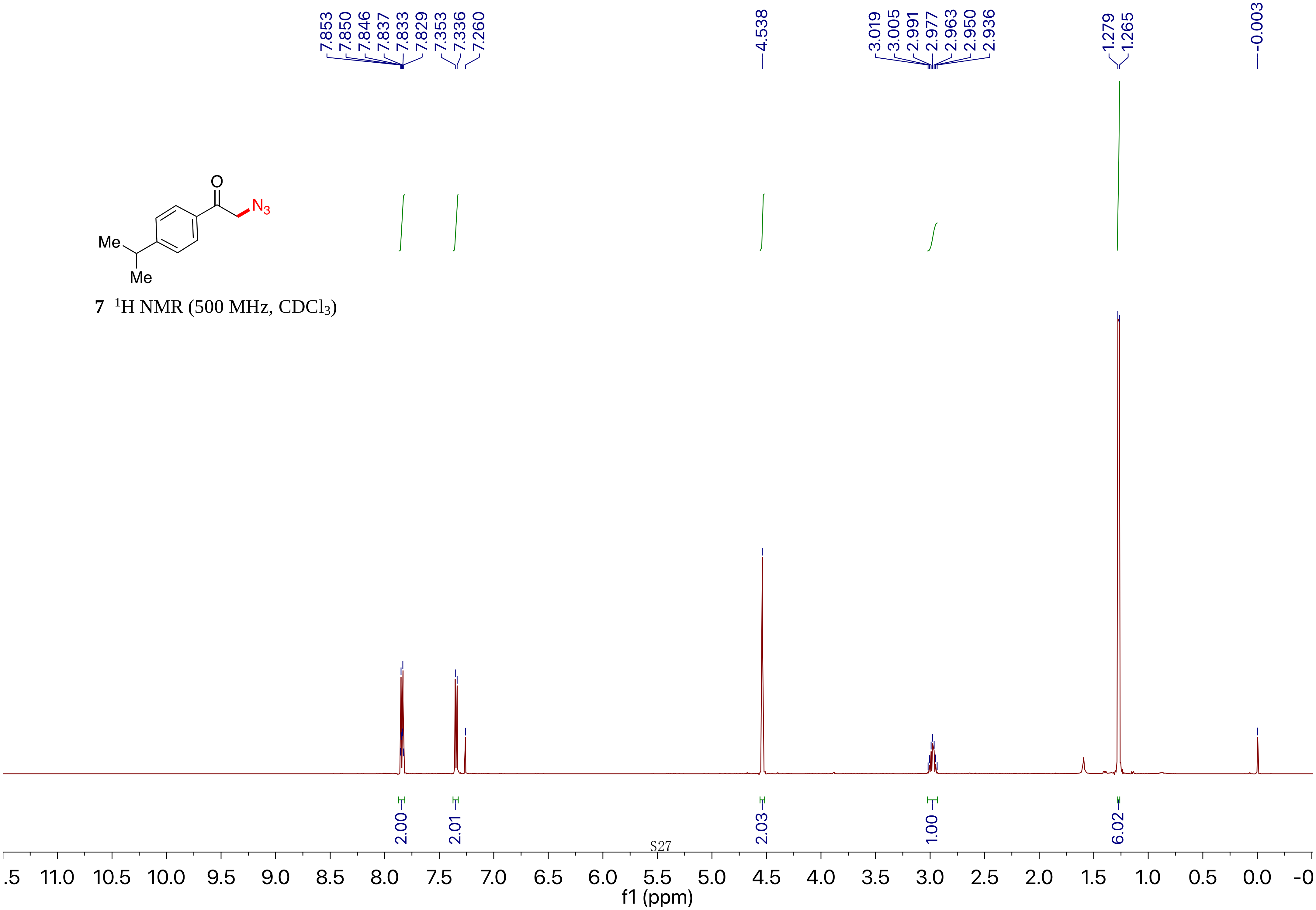

7 ^{1}H NMR (500 MHz, $CDCl_3$)

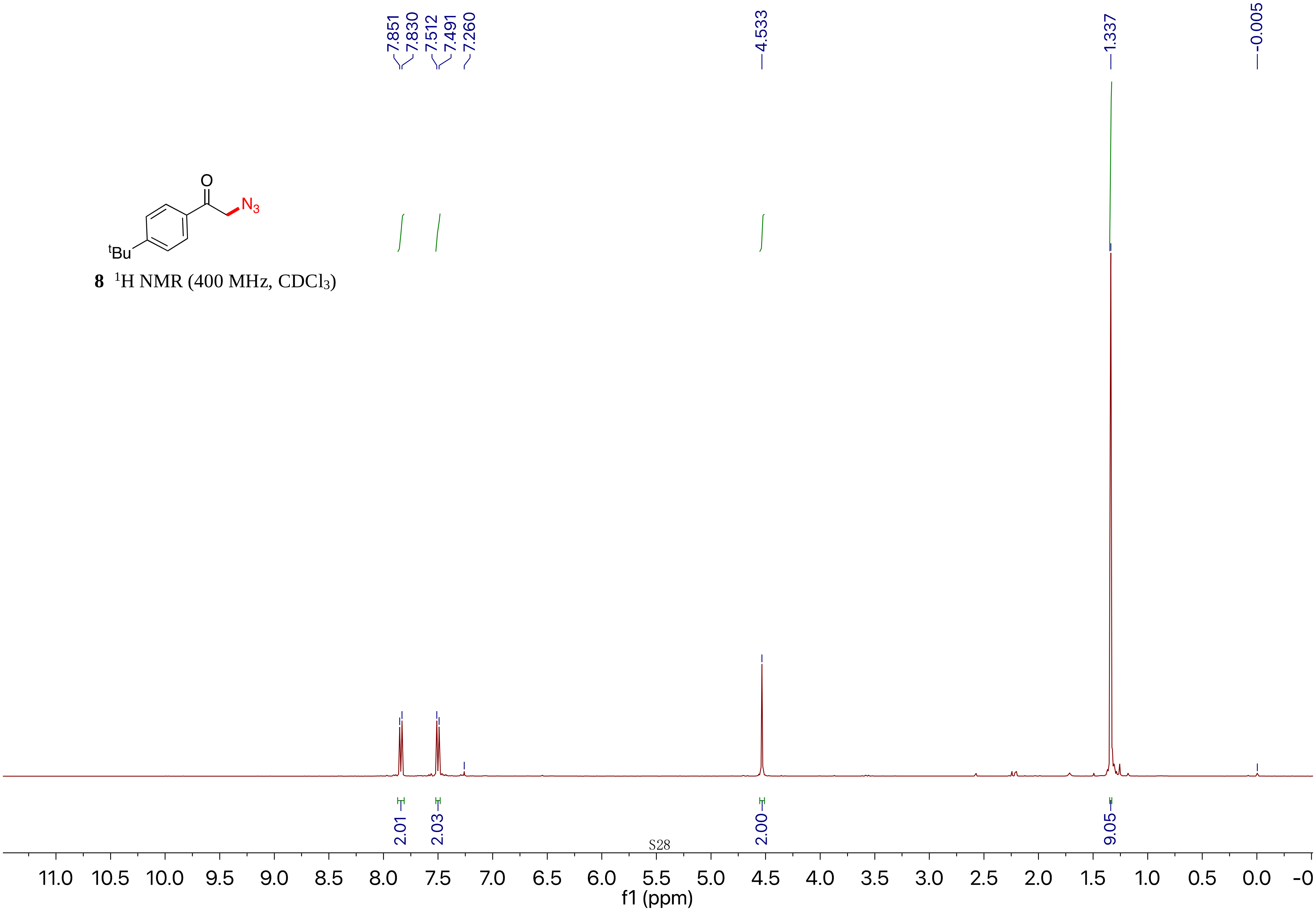

8 ^{1}H NMR (400 MHz, $CDCl_3$)

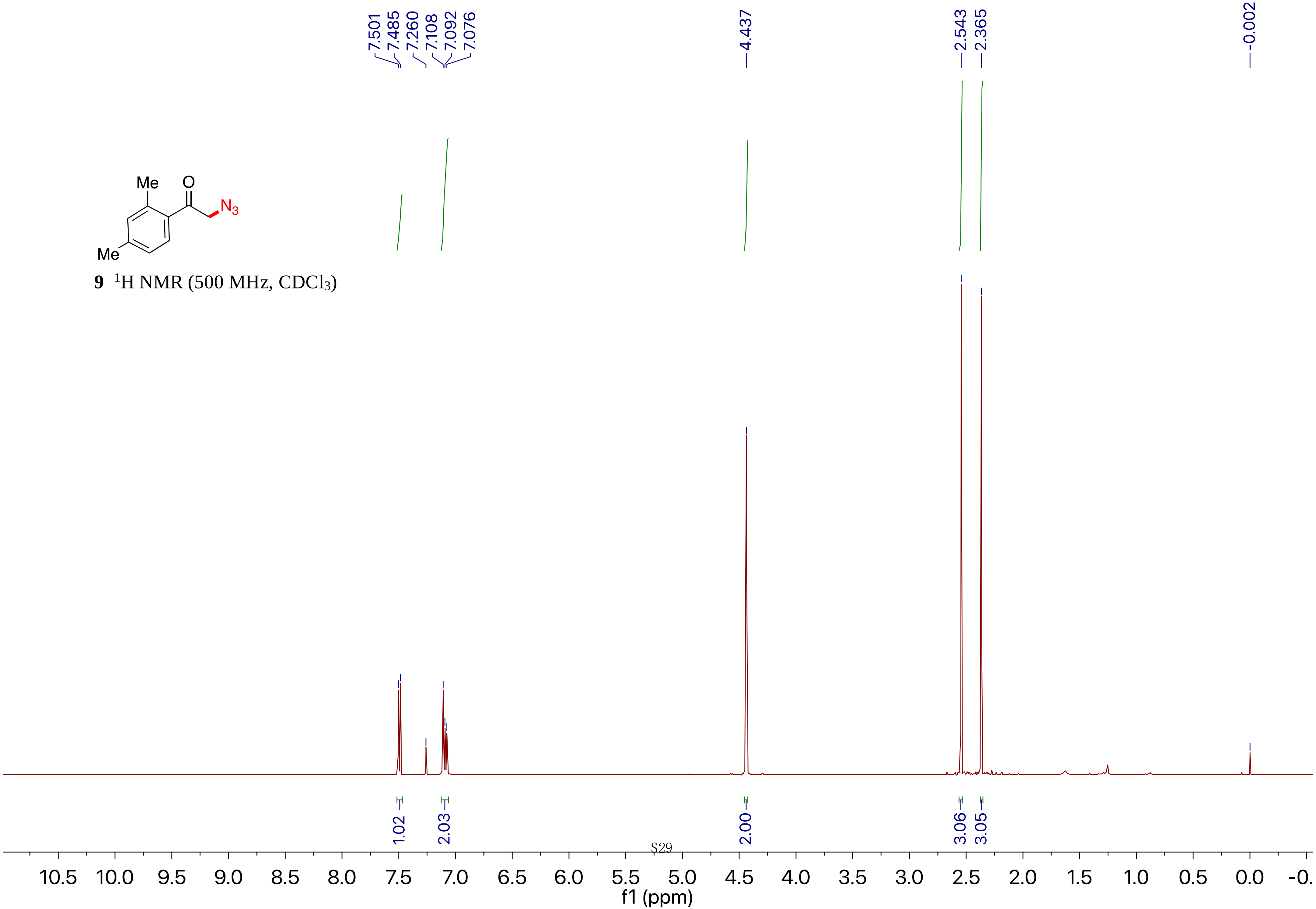

9 ^{1}H NMR (500 MHz, $CDCl_3$)

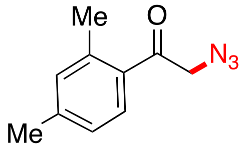

9 ^{13}C NMR (126 MHz, $CDCl_3$)

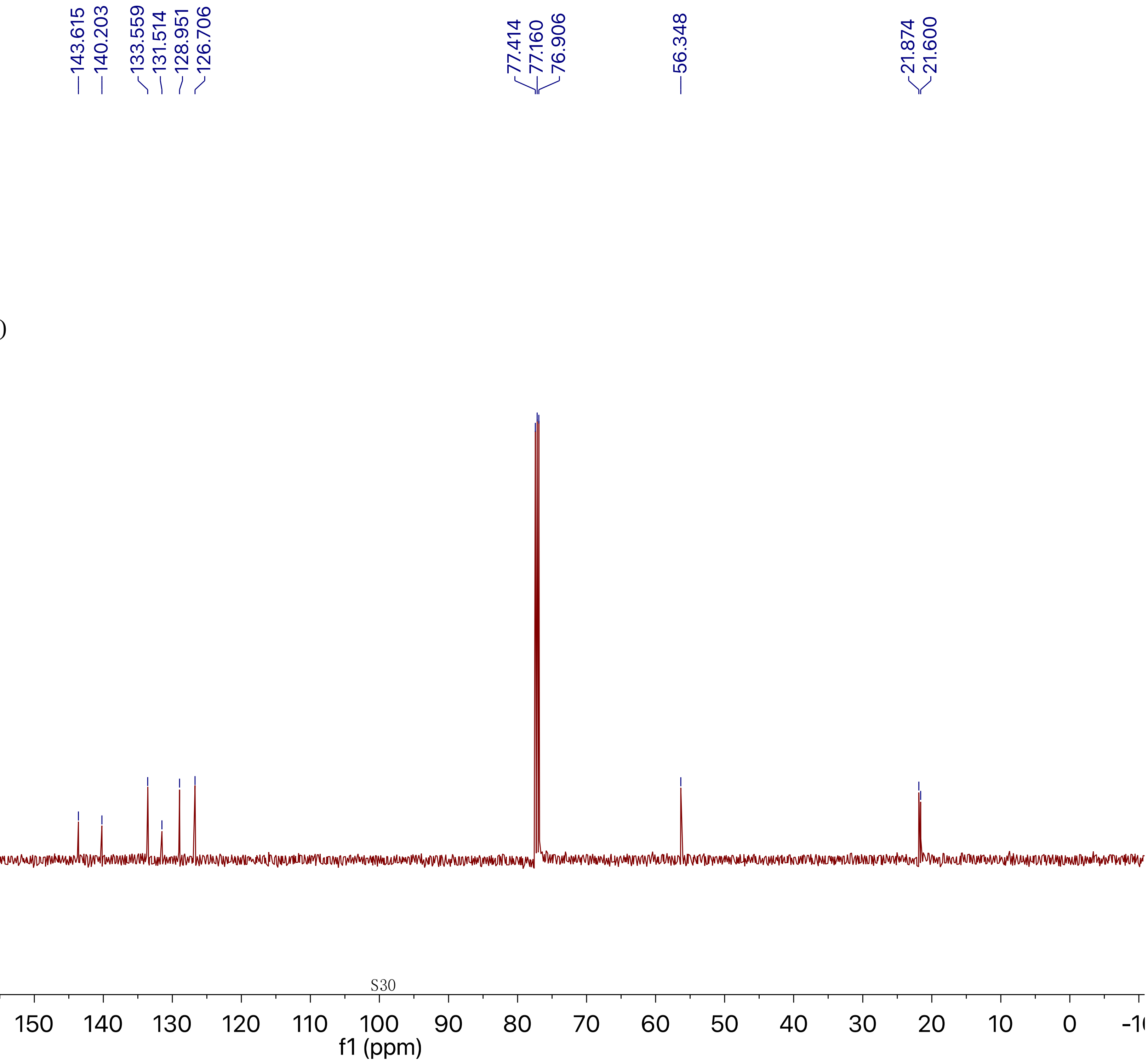

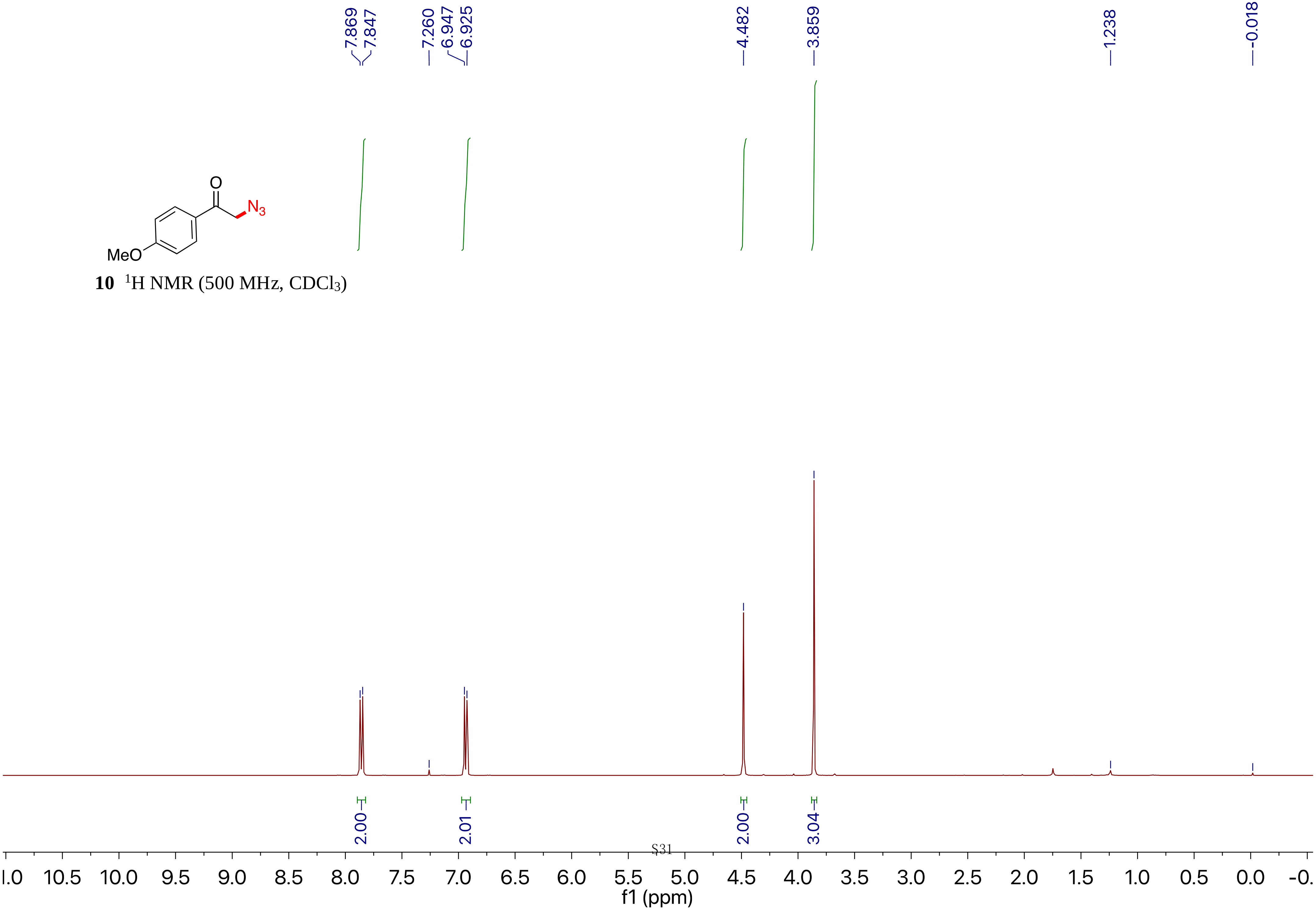

10 ^{1}H NMR (500 MHz, $CDCl_3$)

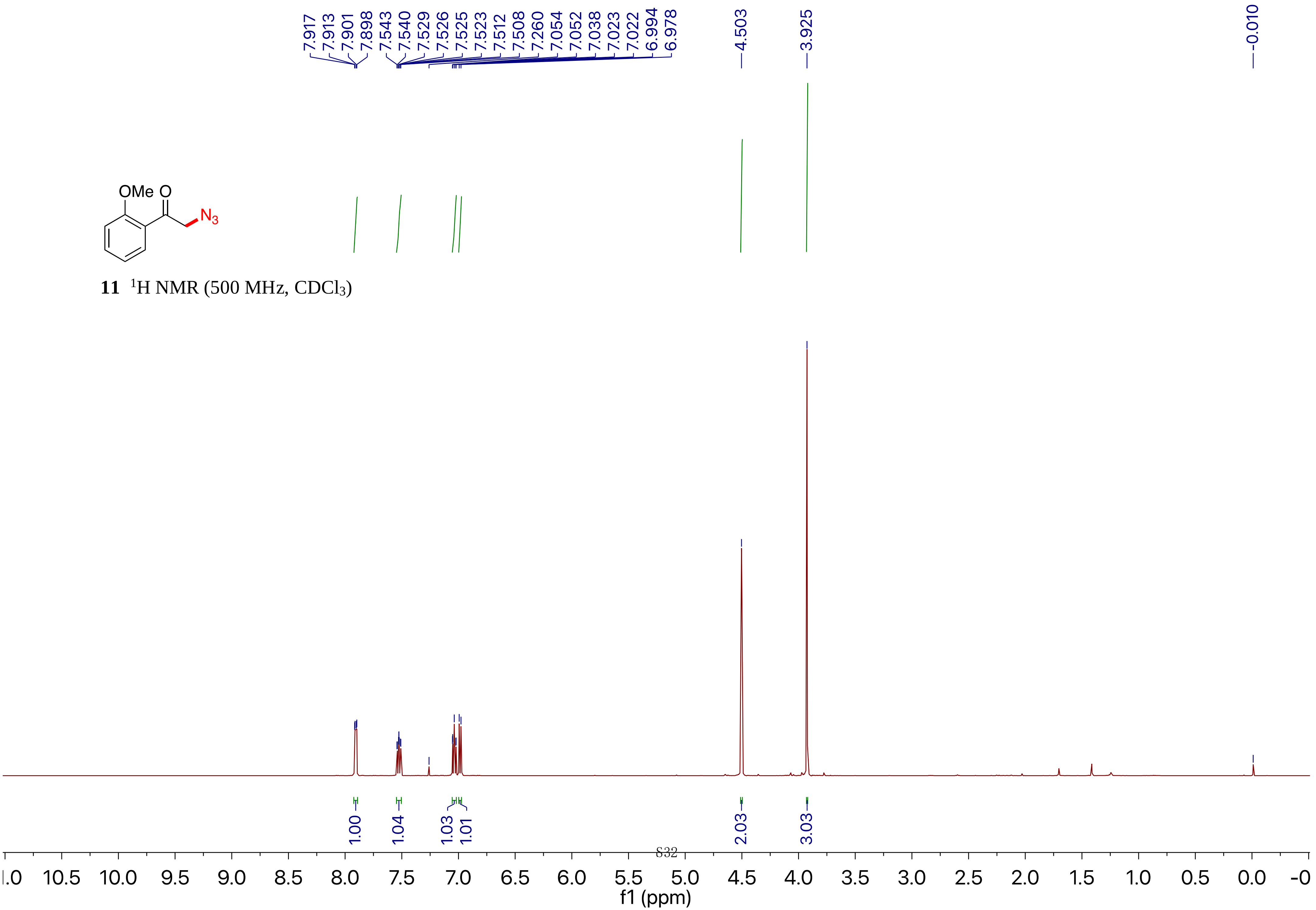
7.917
7.913
7.901
7.898
7.543
7.540
7.529
7.526
7.525
7.523
7.512
7.508
7.260
7.054
7.052
7.038
7.023
7.022
6.994
6.978
4.503
3.925
-0.010
OMe O
N3
11 1H NMR (500 MHz, CDCl3)
1.00
1.04
1.03
1.01
2.03
3.03
11.0 10.5 10.0 9.5 9.0 8.5 8.0 7.5 7.0 6.5 6.0 5.5 5.0 4.5 4.0 3.5 3.0 2.5 2.0 1.5 1.0 0.5 0.0 -0
f1 (ppm)

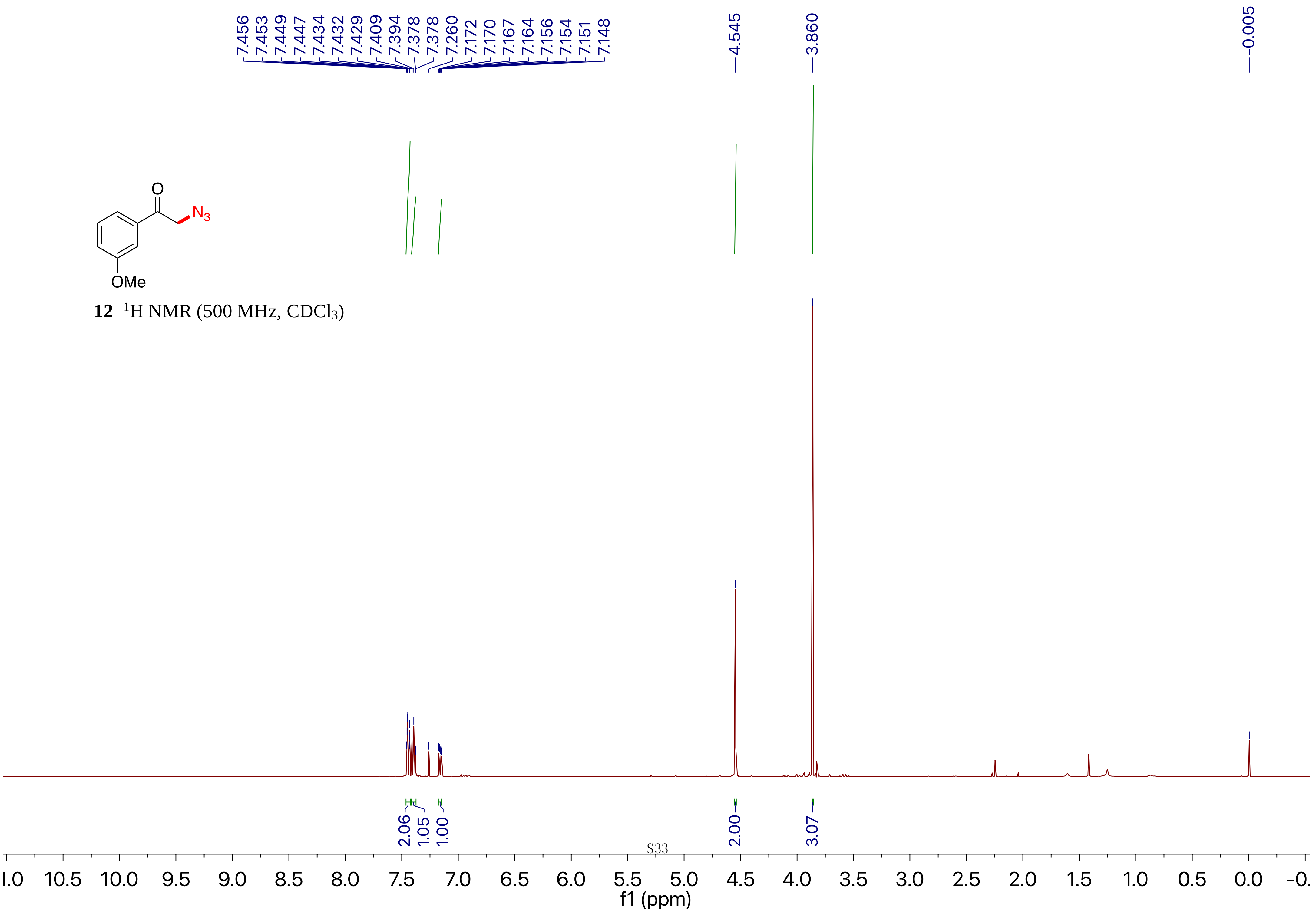

12 ^{1}H NMR (500 MHz, $CDCl_3$)

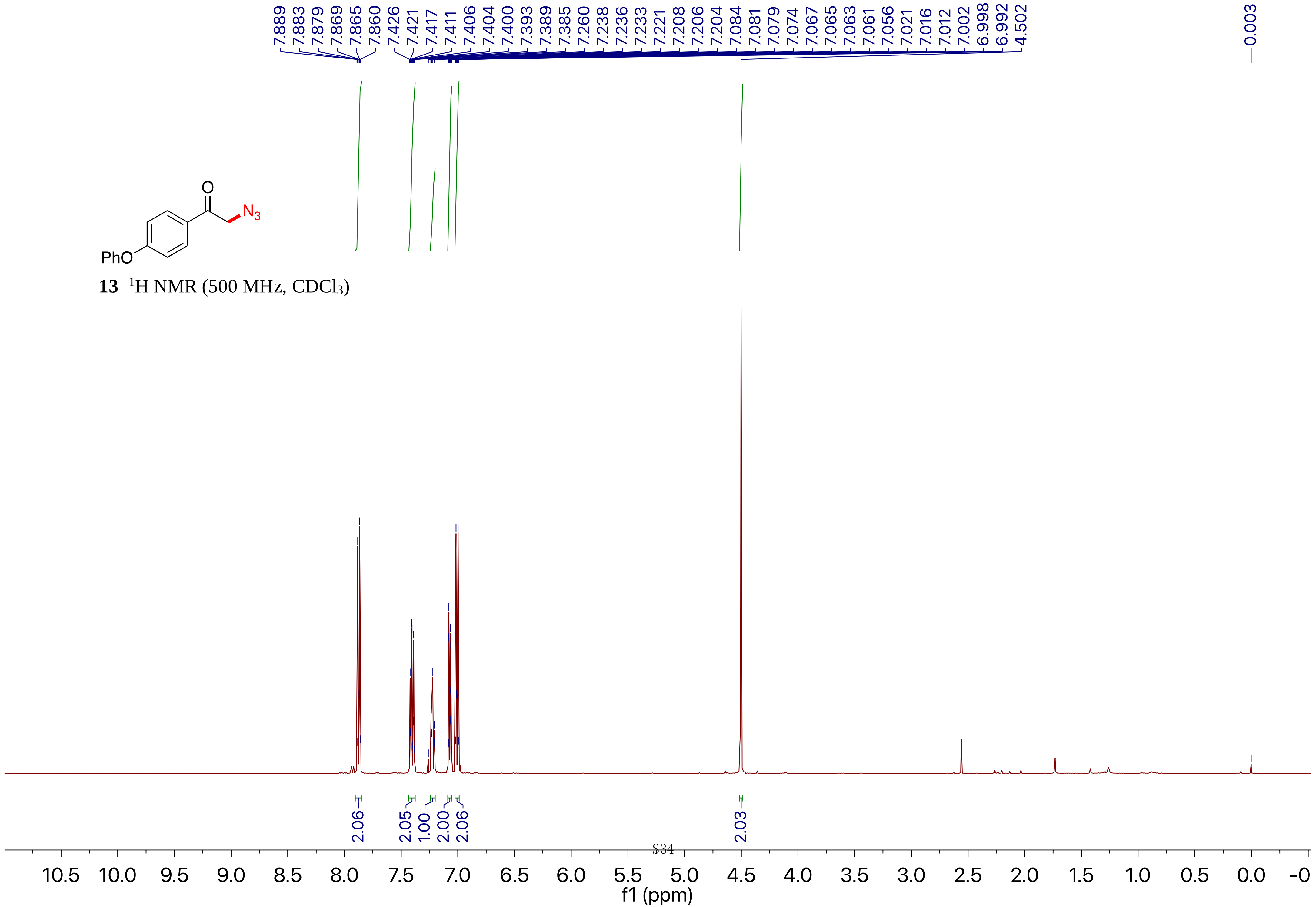

13 ^{1}H NMR (500 MHz, $CDCl_3$)

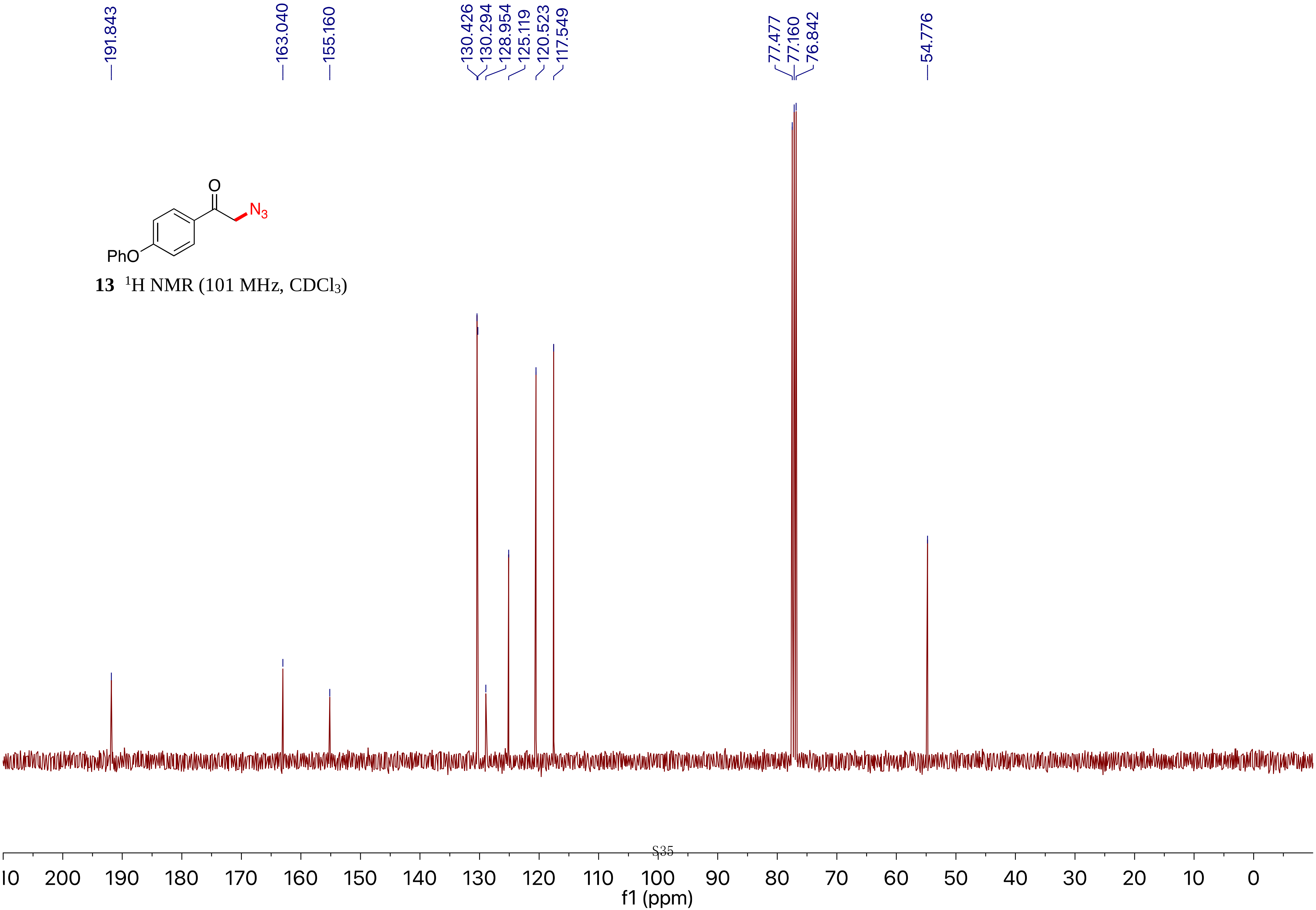
191.843
163.040
155.160
130.426
130.294
128.954
125.119
120.523
117.549
77.477
77.160
76.842
54.776
O
N_3
PhO
13 ^{1}H NMR (101 MHz, $CDCl_3$)
f1 (ppm)

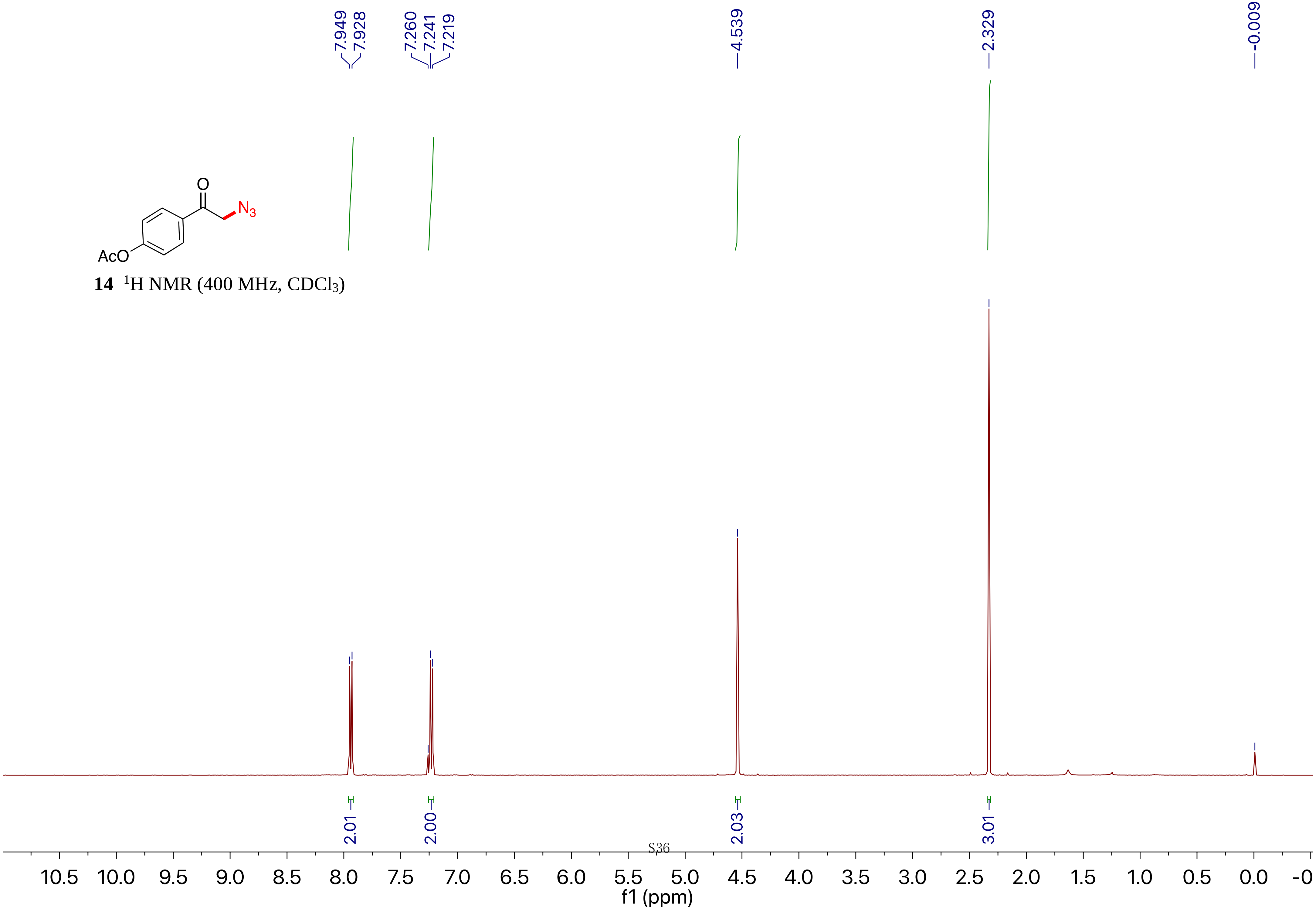

14 ^{1}H NMR (400 MHz, $CDCl_3$)

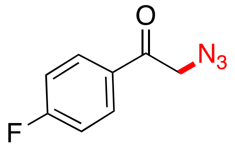

15 ^{1}H NMR (400 MHz, $CDCl_3$)

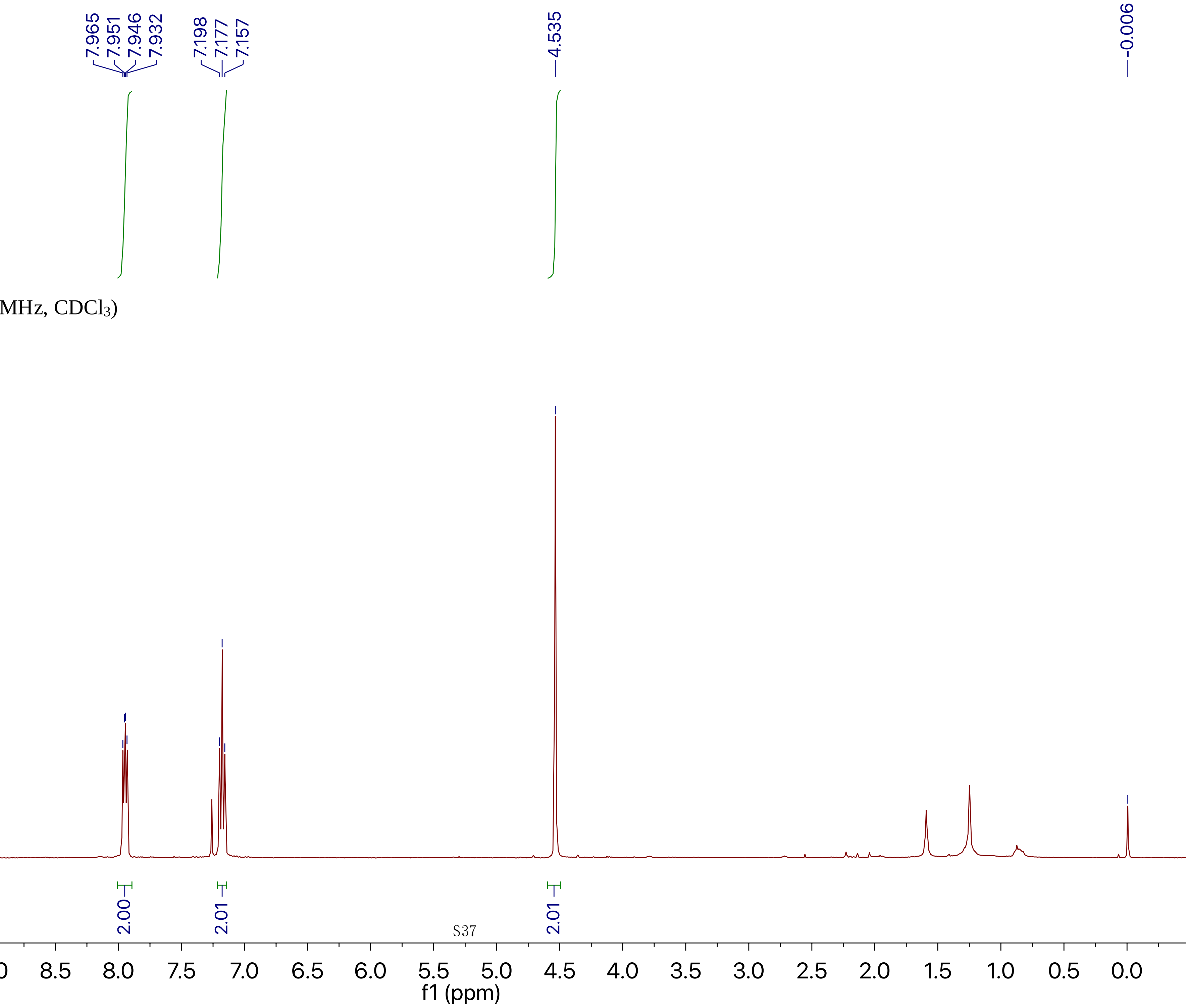

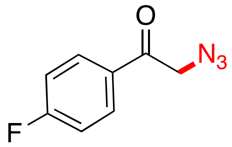

15 ^{19}F NMR (471 MHz, $CDCl_3$)

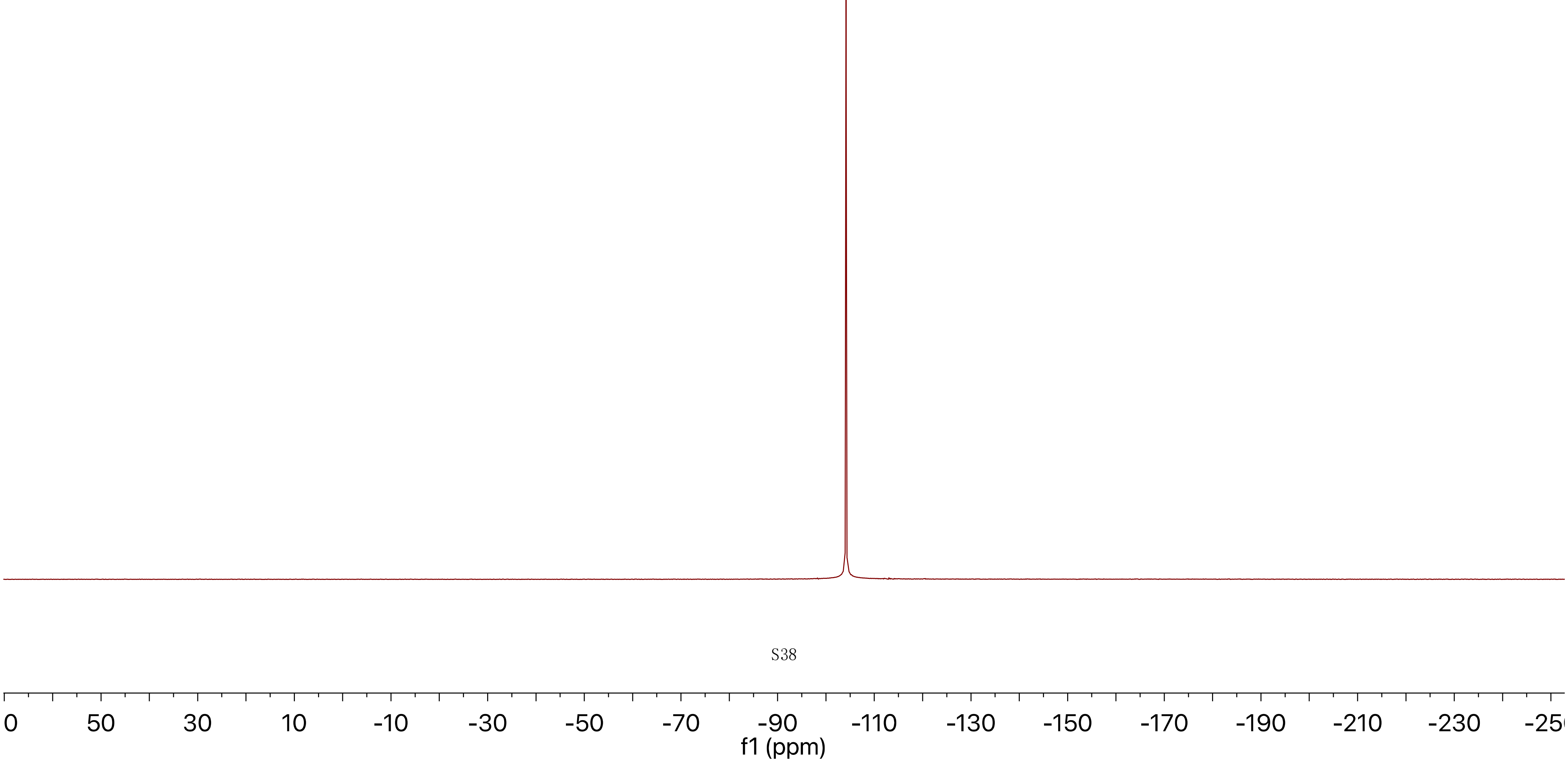

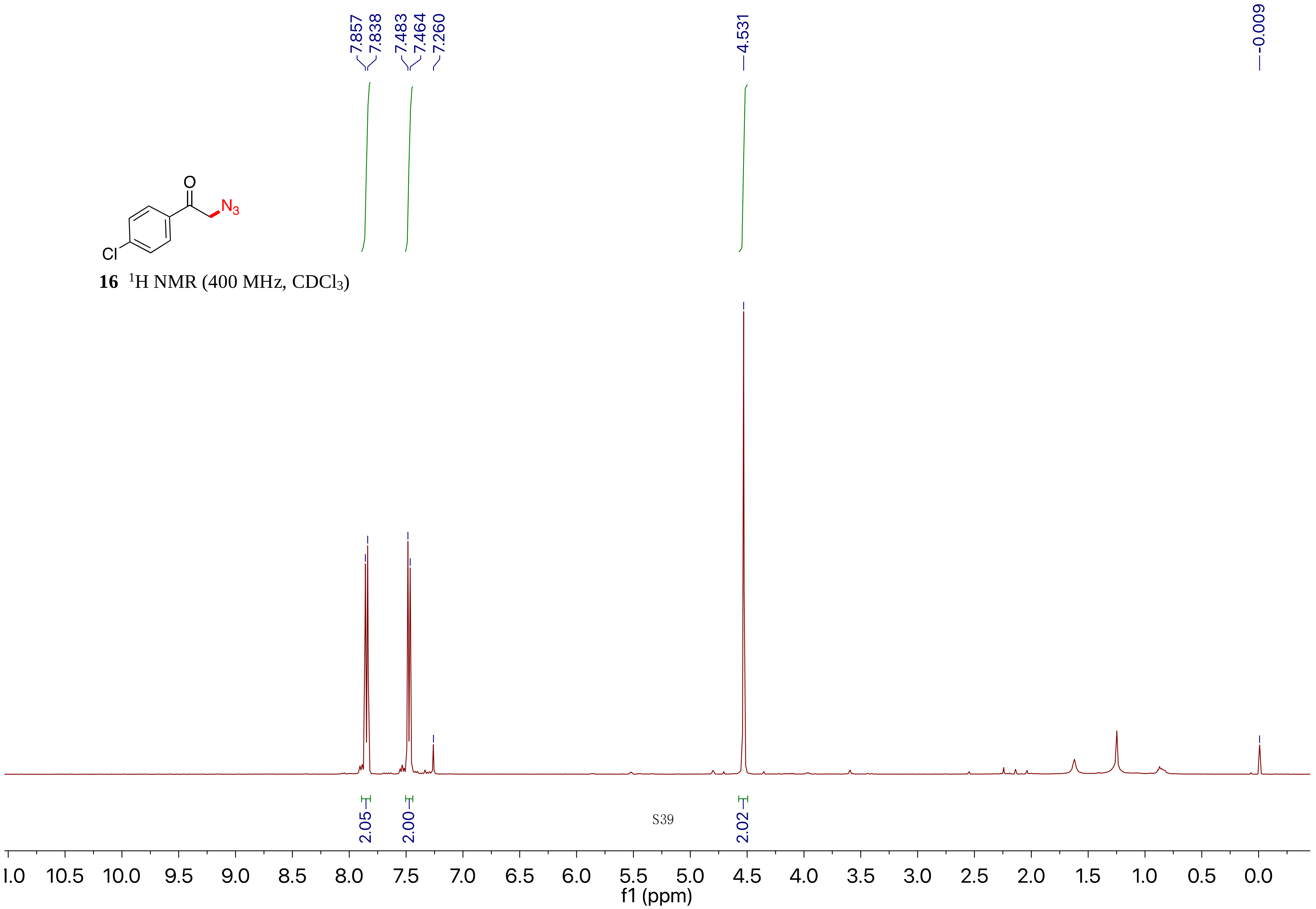

16 ^{1}H NMR (400 MHz, $CDCl_3$)

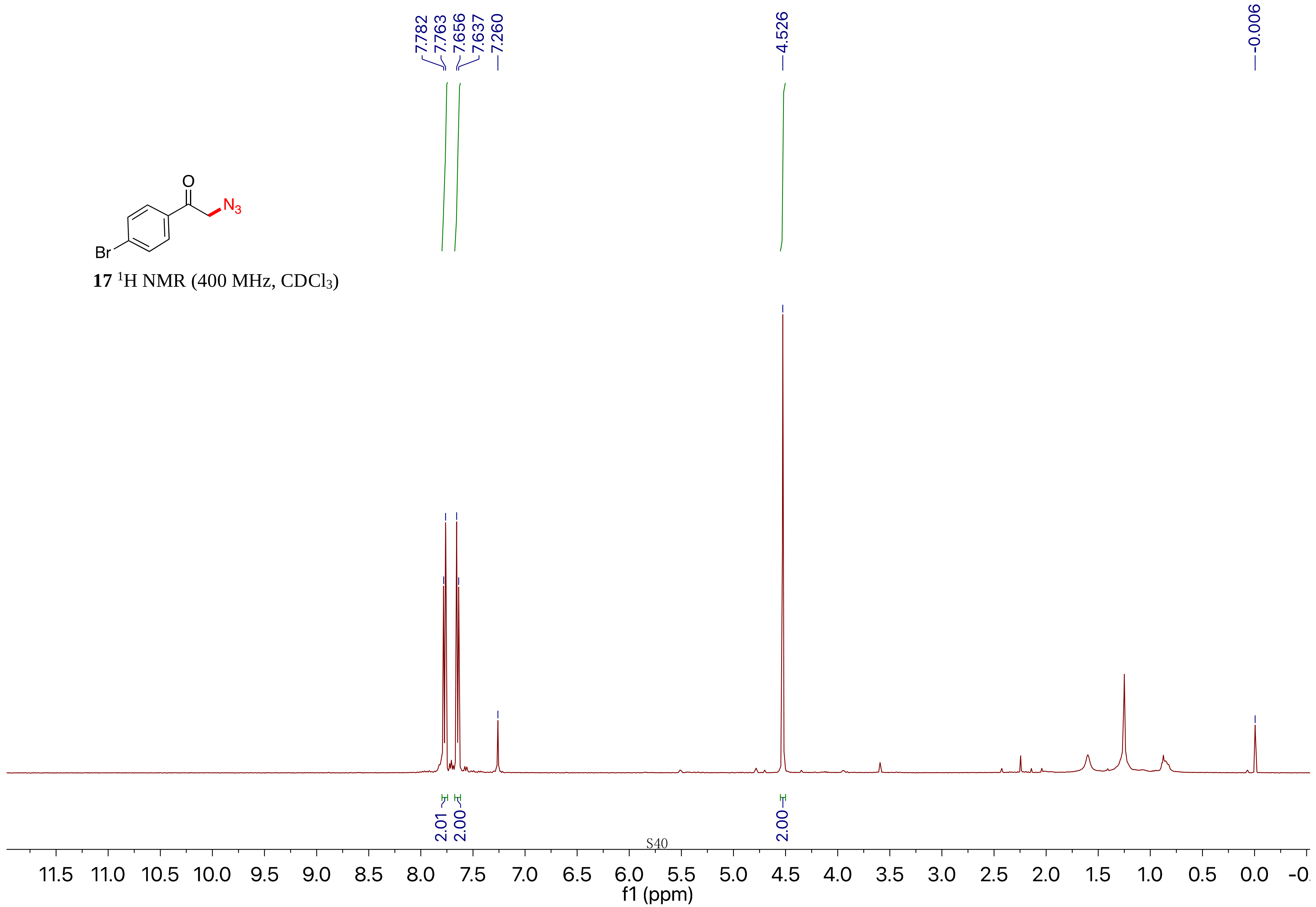

17 ^{1}H NMR (400 MHz, $CDCl_3$)

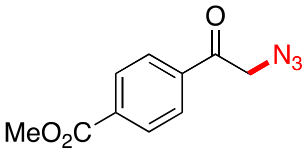

18 ^{1}H NMR (400 MHz, $CDCl_3$)

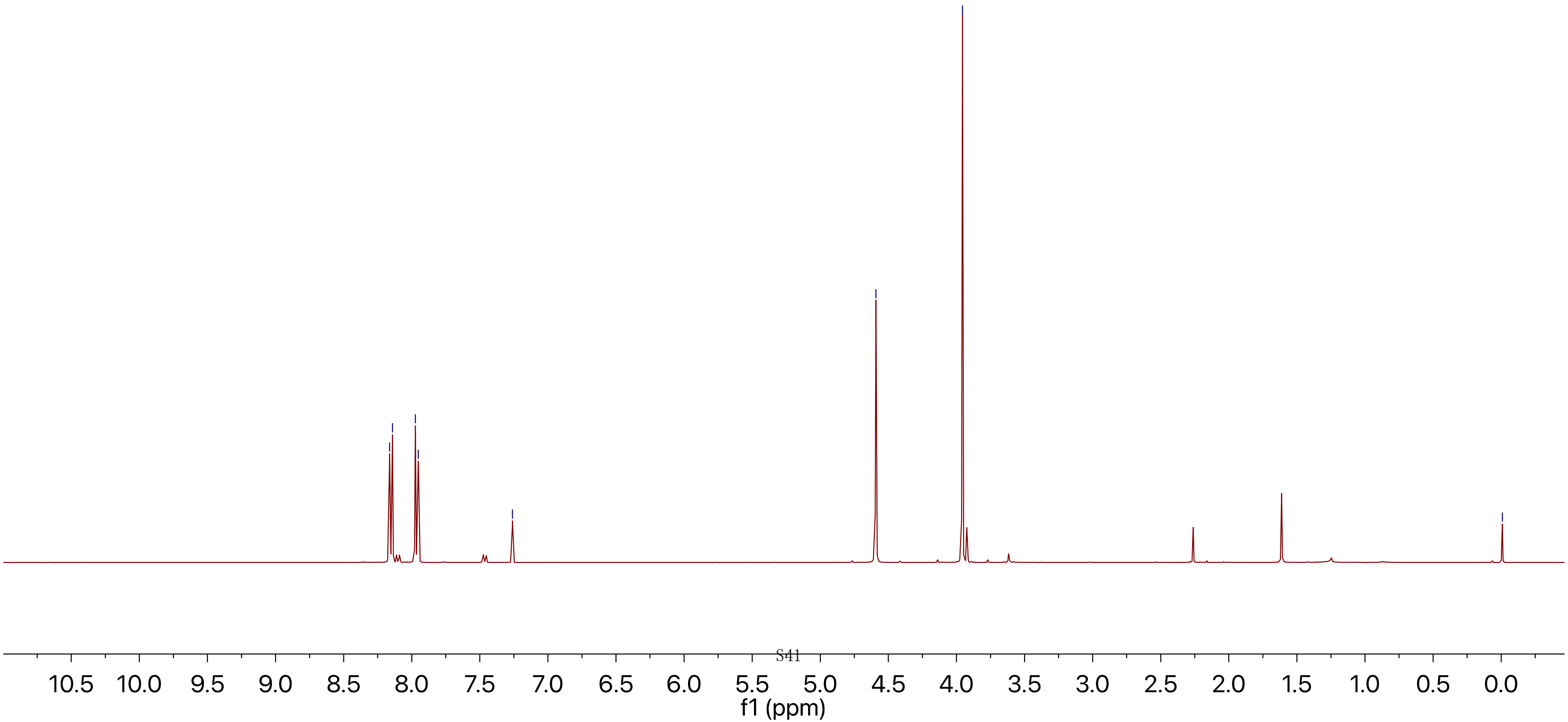

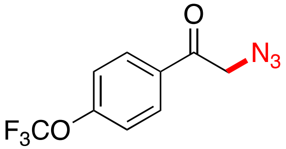

19 ^{1}H NMR (400 MHz, $CDCl_3$)

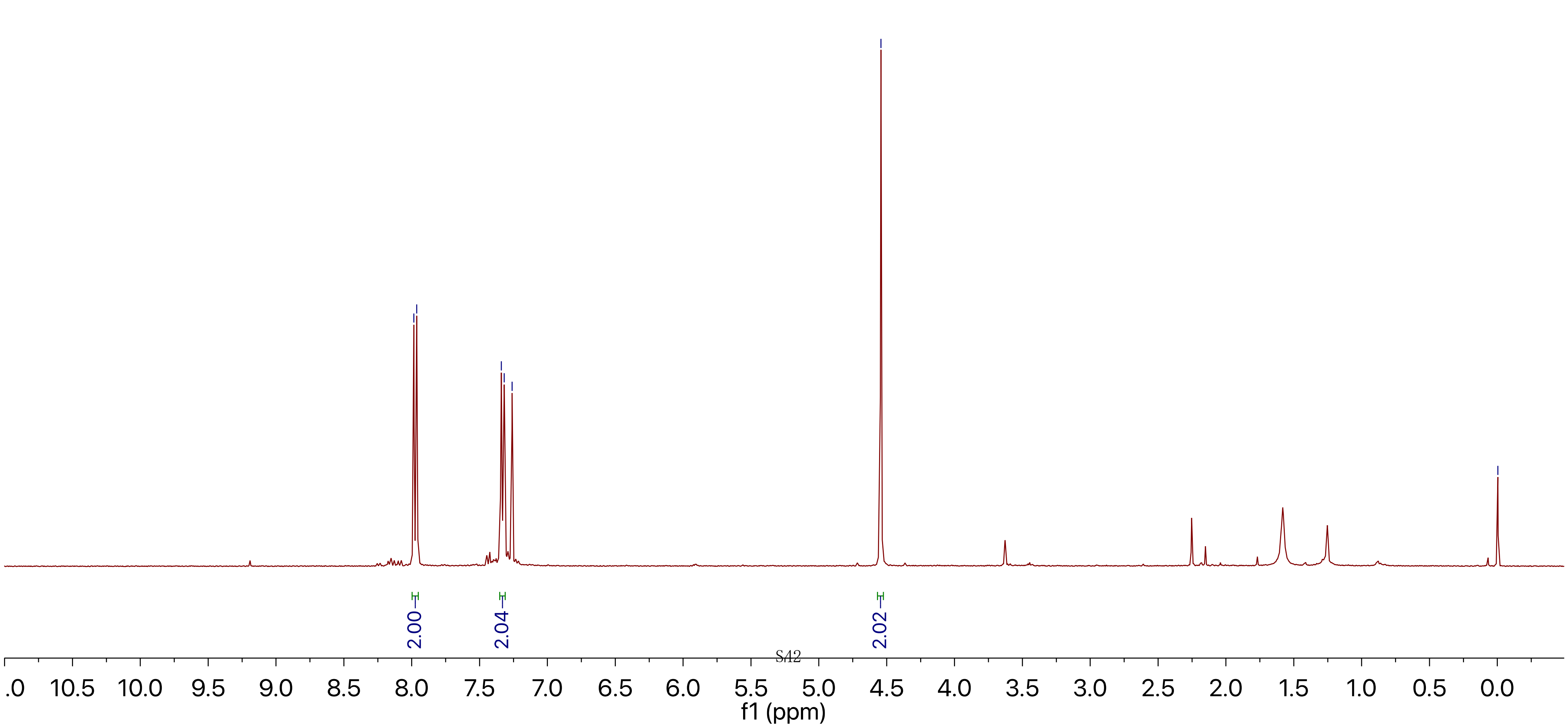

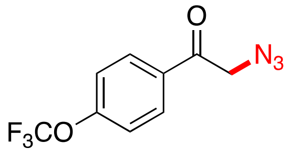

19 ^{19}F NMR (471 MHz, $CDCl_3$)

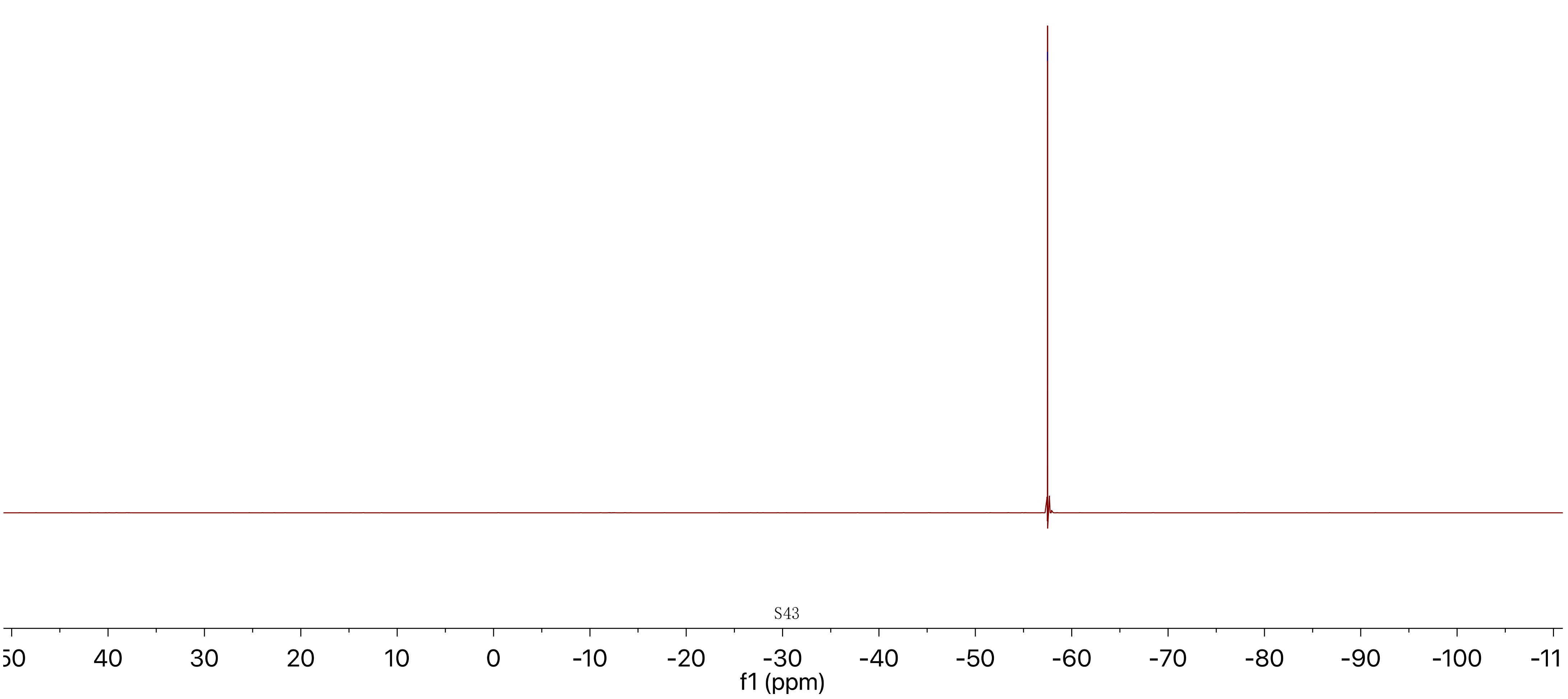

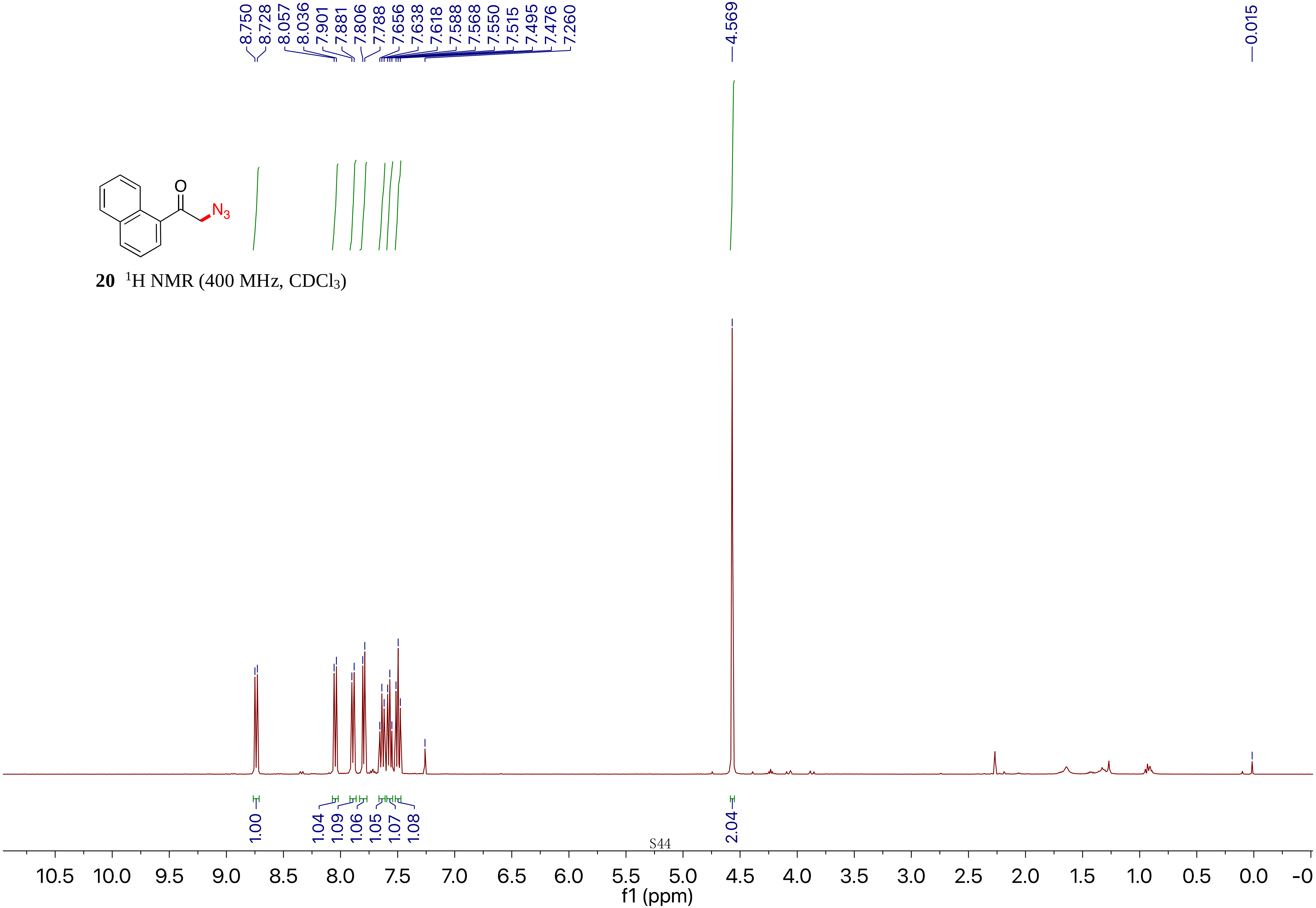

20 ^{1}H NMR (400 MHz, $CDCl_3$)

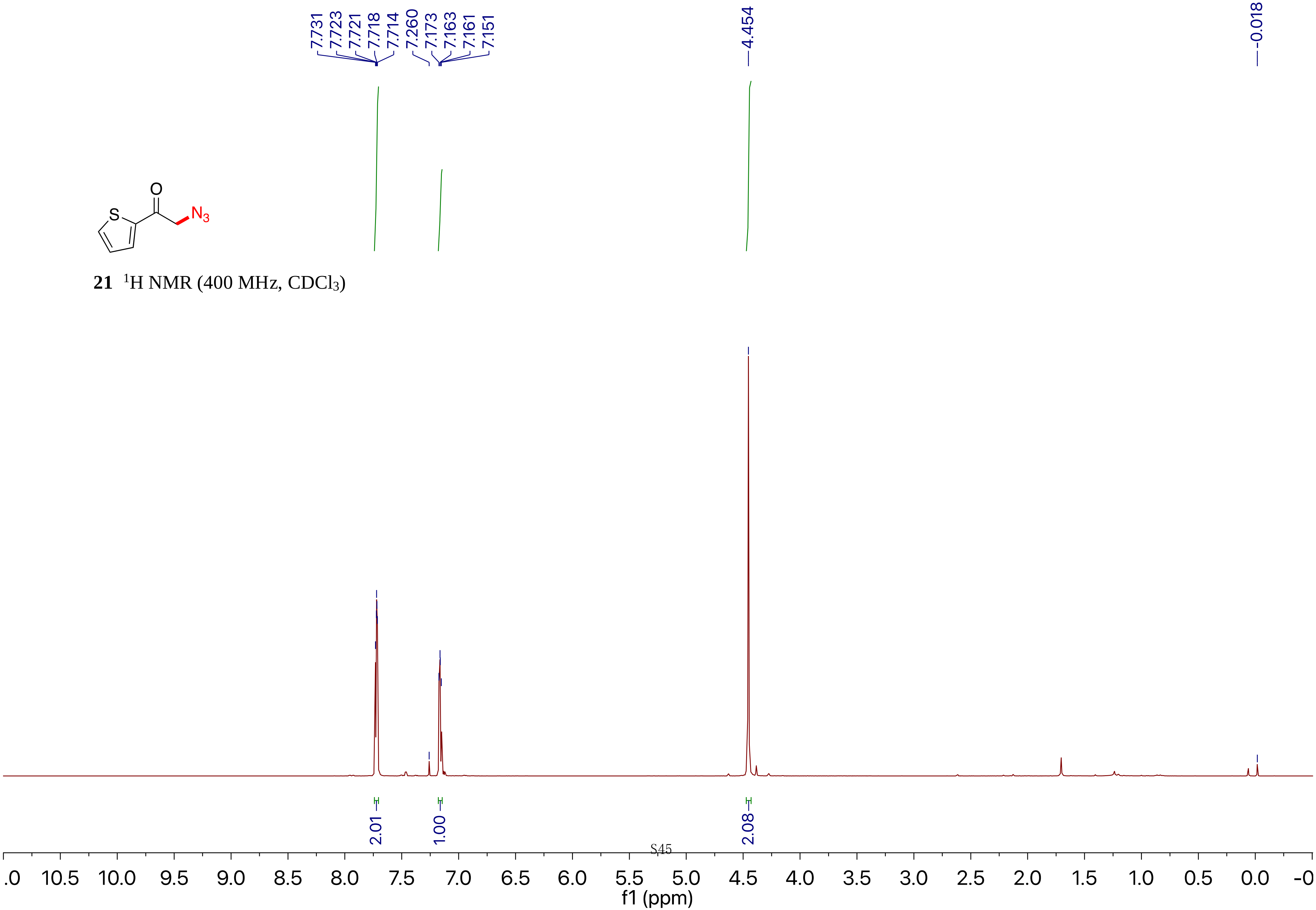

21 ^{1}H NMR (400 MHz, $CDCl_3$)

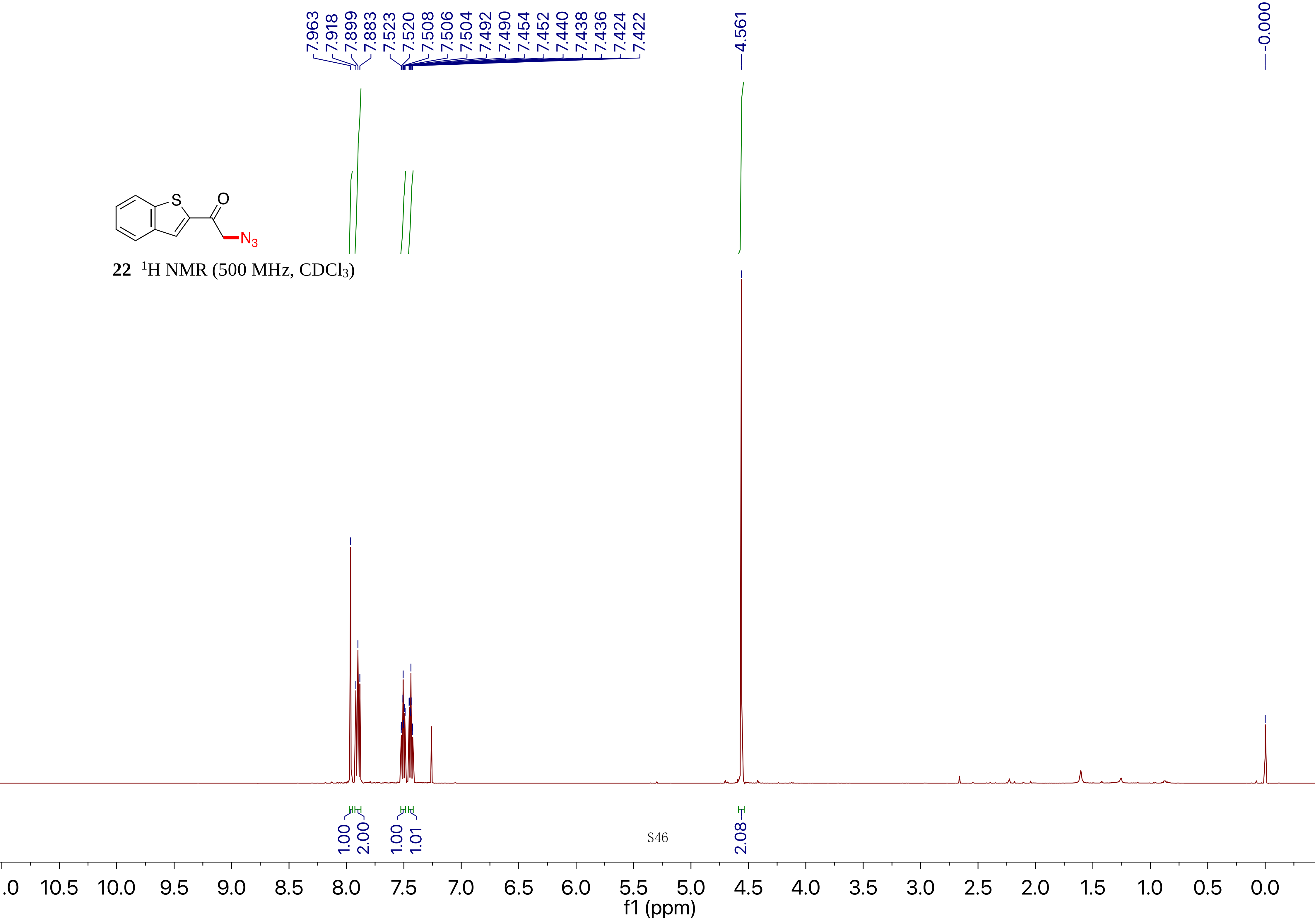
7.963
7.918
7.899
7.883
7.523
7.520
7.508
7.506
7.504
7.492
7.490
7.454
7.452
7.440
7.438
7.436
7.424
7.422
4.561
-0.000
S
O
N3
22 1H NMR (500 MHz, CDCl3)
1.00
2.00
1.00
1.01
2.08
f1 (ppm)

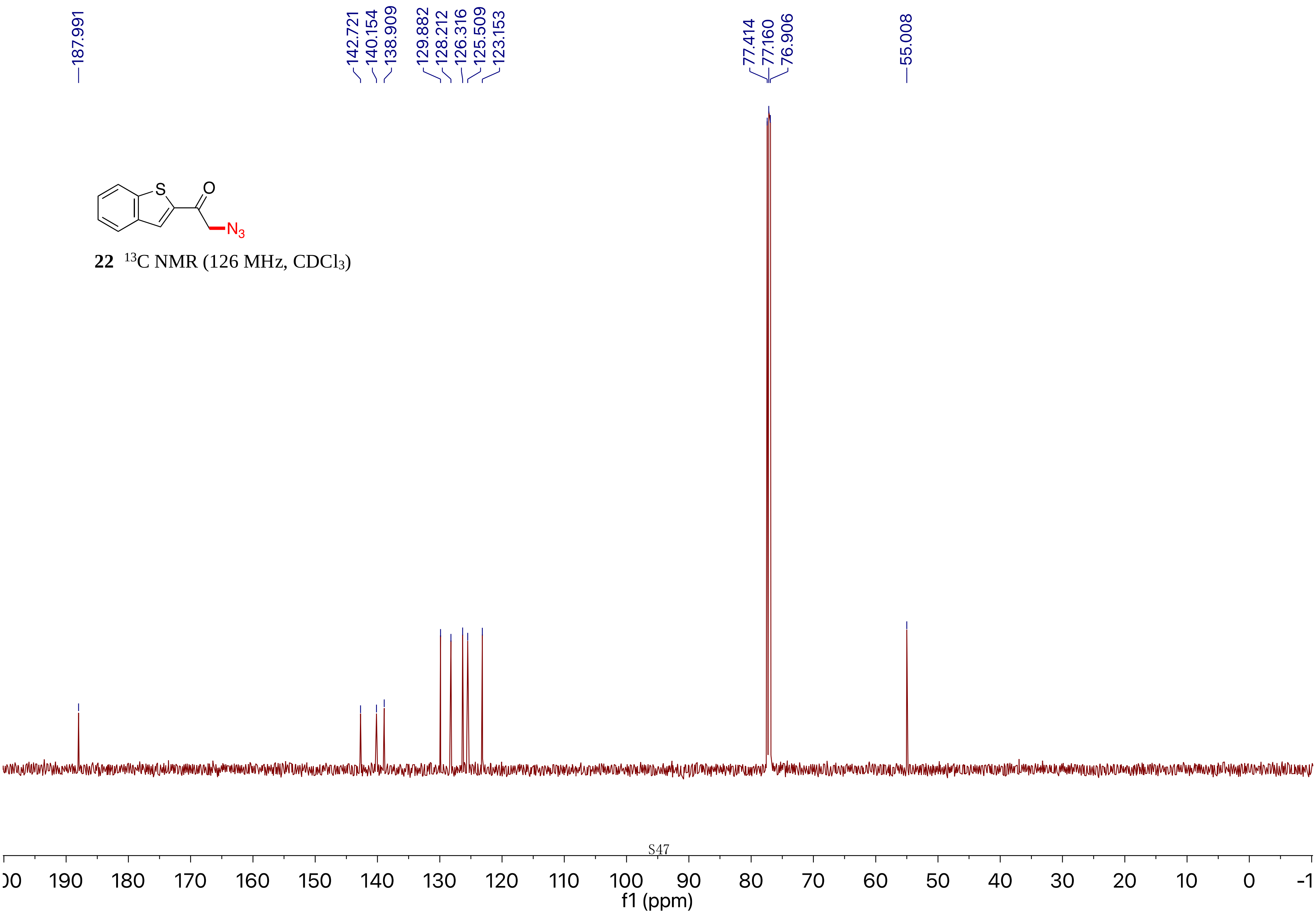

22 ^{13}C NMR (126 MHz, $CDCl_3$)

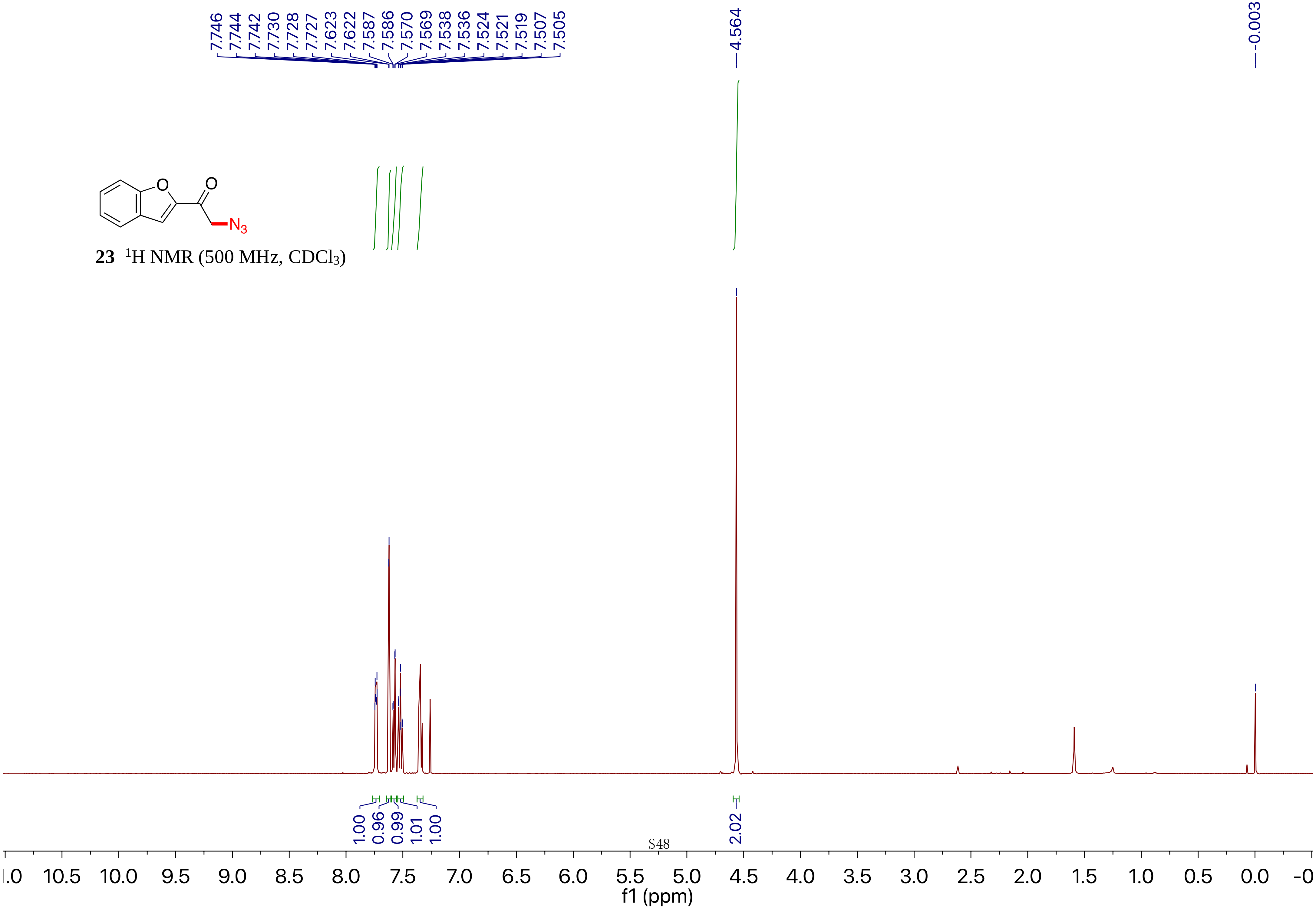

23 ^{1}H NMR (500 MHz, $CDCl_3$)

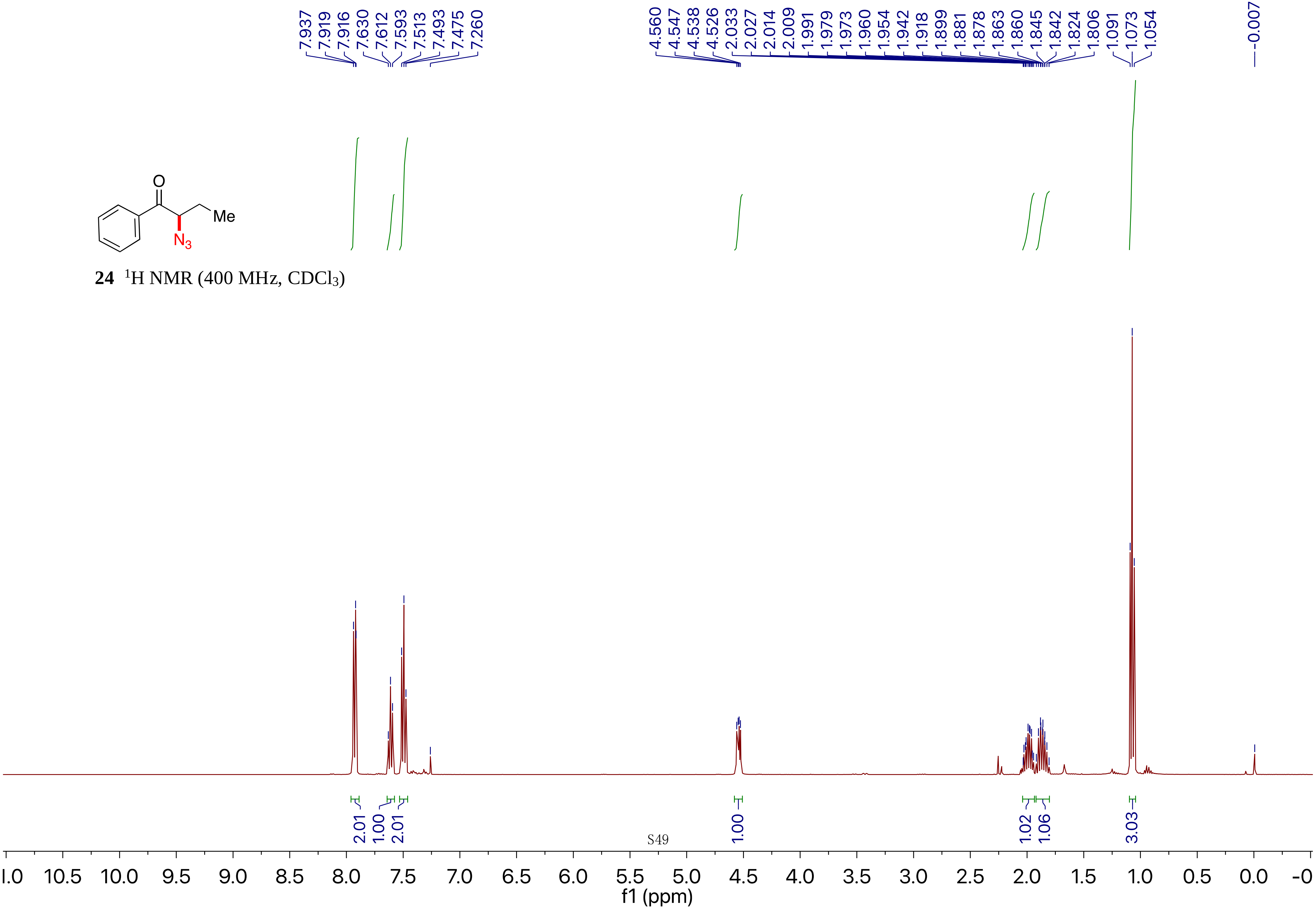

24 ^{1}H NMR (400 MHz, $CDCl_3$)

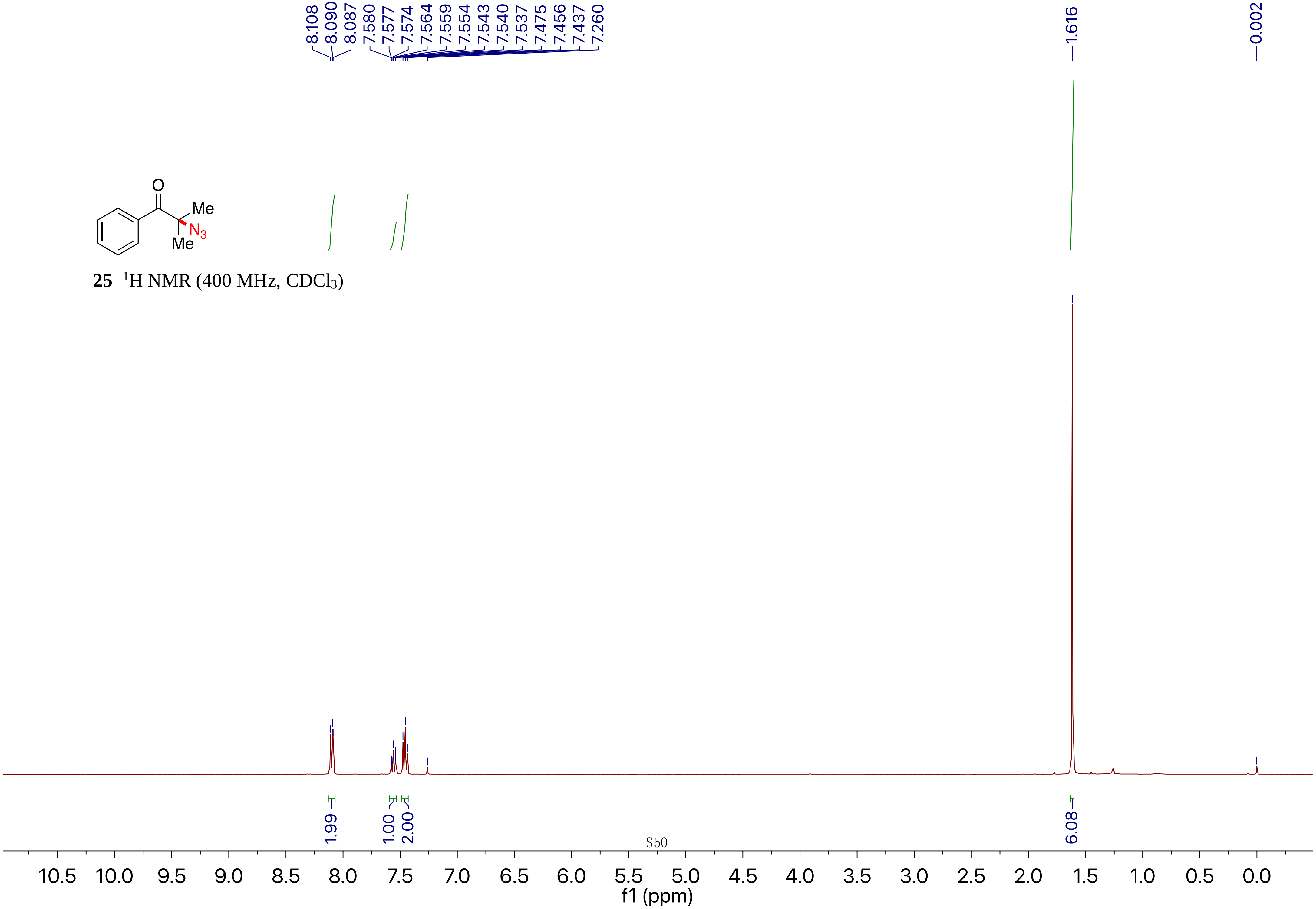

25 ^{1}H NMR (400 MHz, $CDCl_3$)

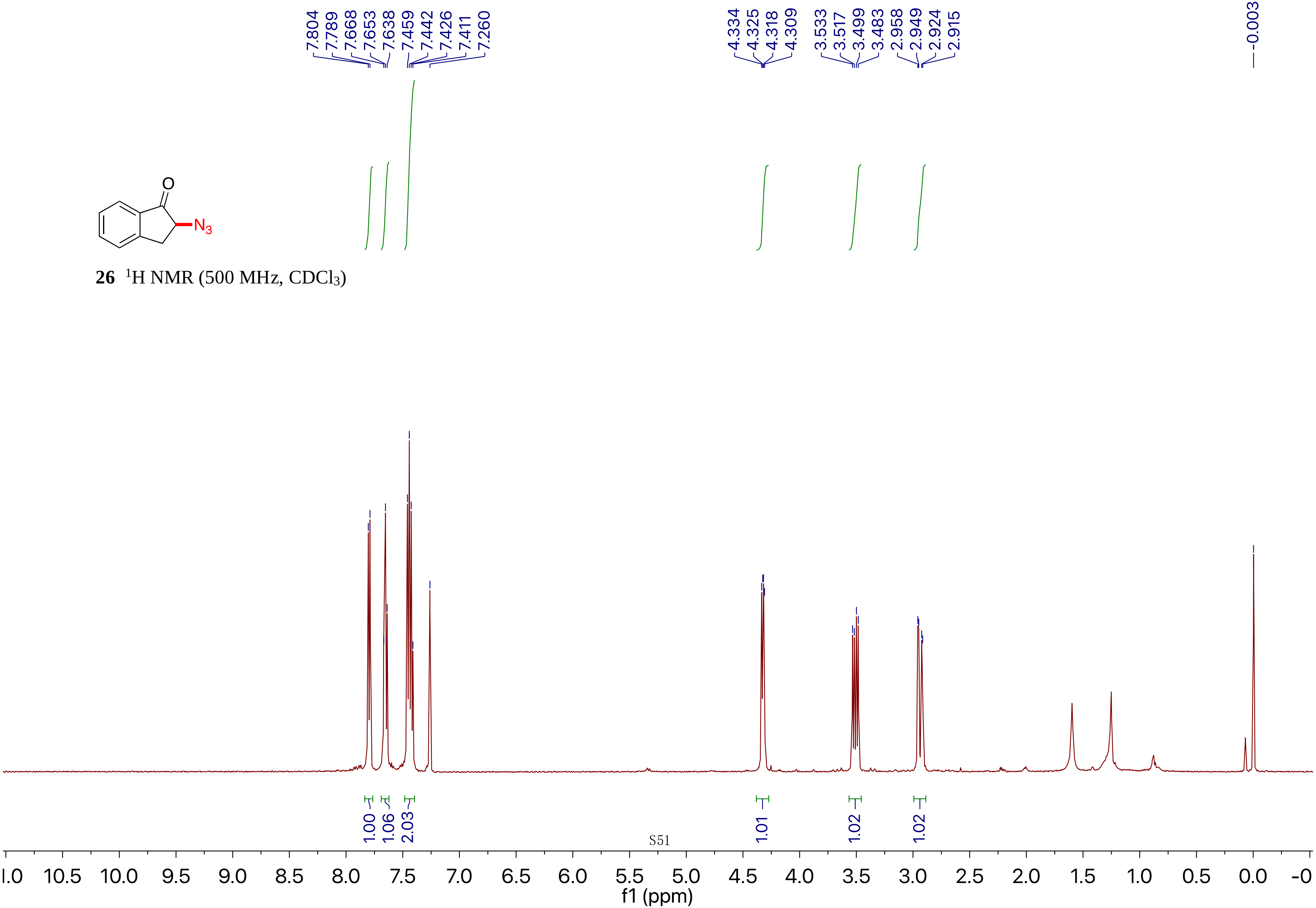

26 ^{1}H NMR (500 MHz, $CDCl_3$)

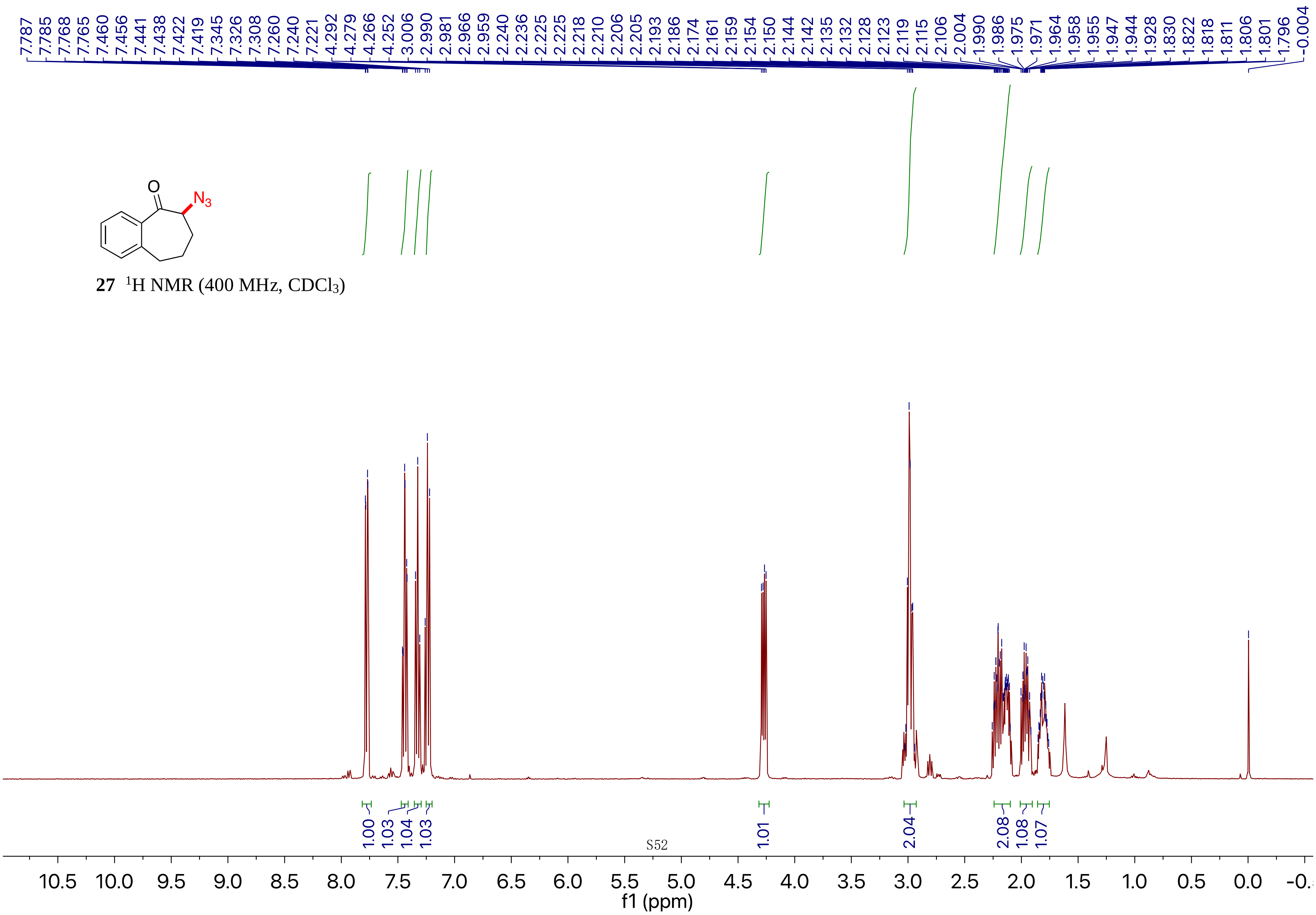

27 ^{1}H NMR (400 MHz, $CDCl_3$)

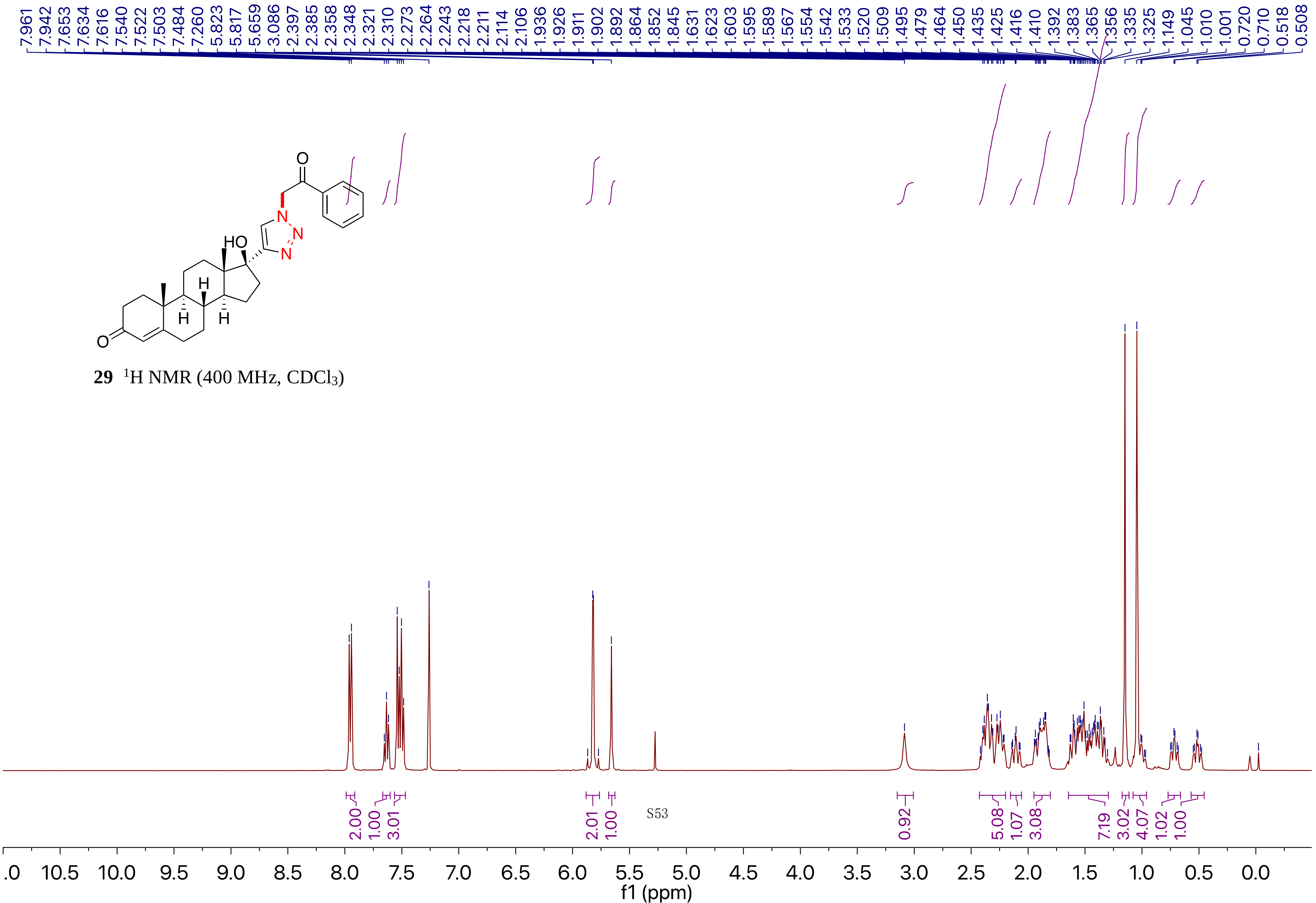

29 ^{1}H NMR (400 MHz, $CDCl_3$)

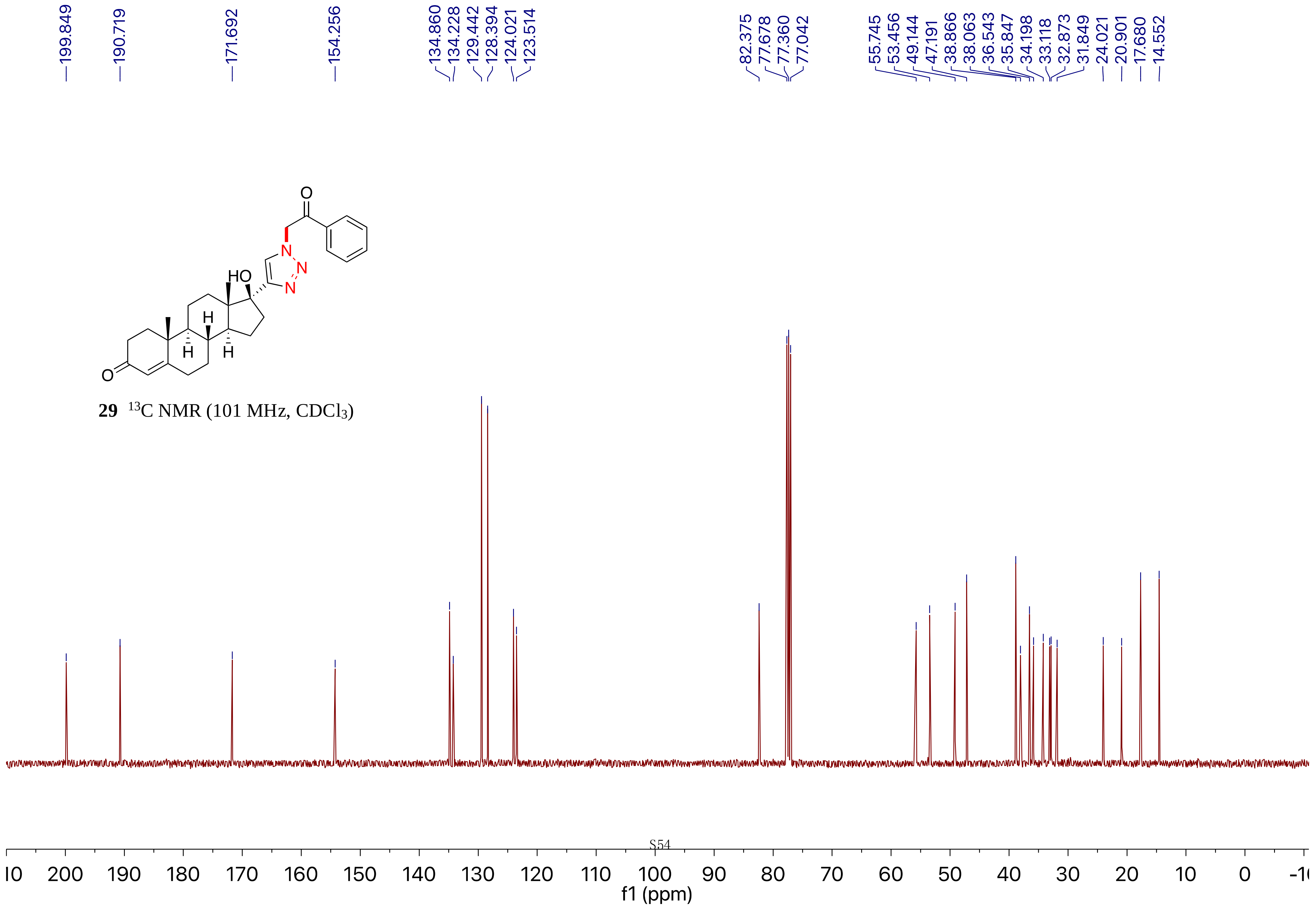

29 ^{13}C NMR (101 MHz, $CDCl_3$)

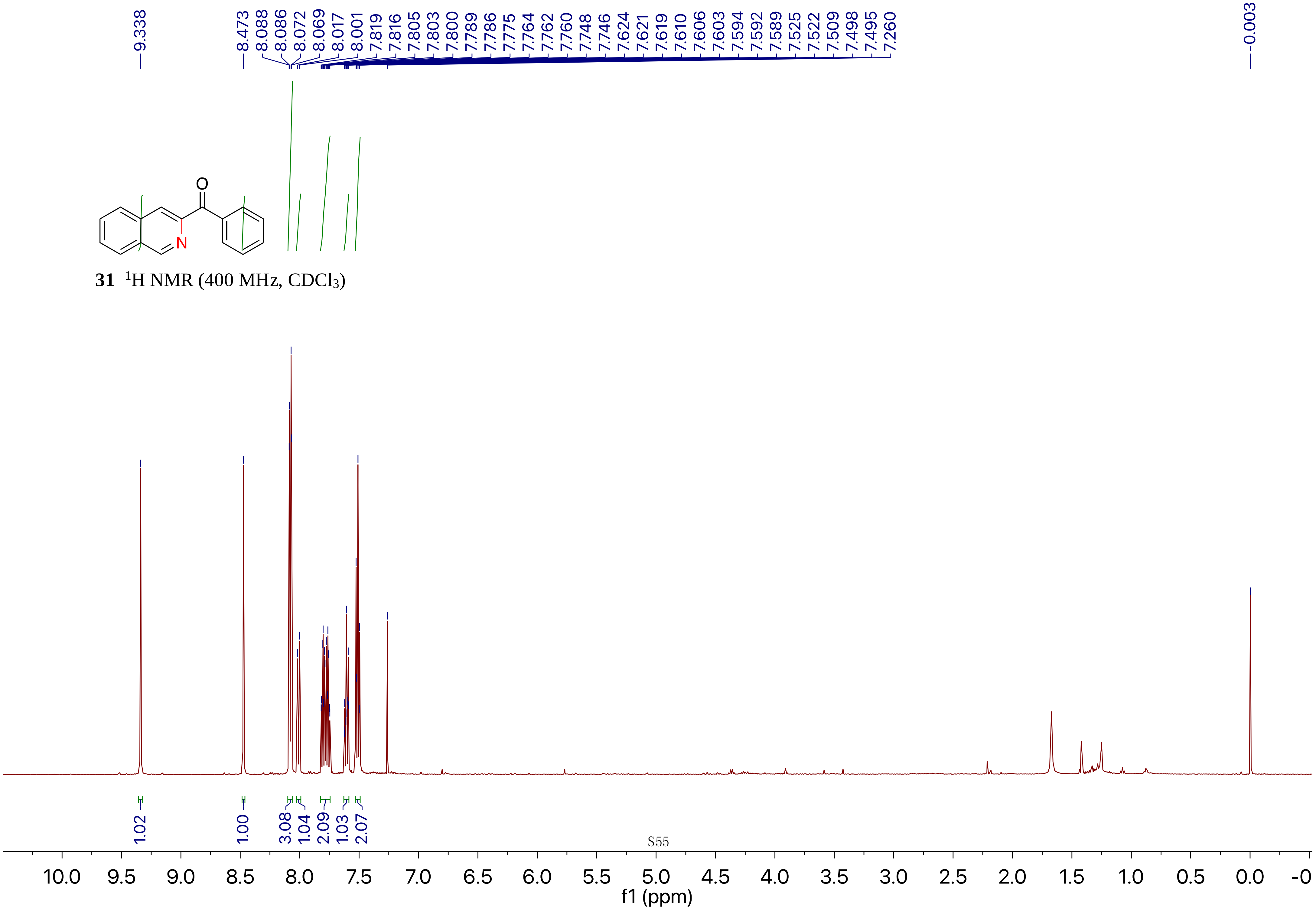

31 ^{1}H NMR (400 MHz, $CDCl_3$)
